# Supplementary material for: Effect of Metal Ions on Hybrid Graphite-Diamond Nanowire Growth: Conductivity Measurements from a Single Nanowire Device
Source: Nanomaterials (Basel). 2019 Mar 11;9(3):415. doi: 10.3390/nano9030415 (PMC6473948; doi:10.3390/nano9030415)
Supplement: Supplementary file 1 [file nanomaterials-09-00415-s001.pdf]

Supporting Information

Effect of Metal Ions on Hybrid Graphite-Diamond Nanowires Growth: Conductivity Measurements from a Single Nanowire Device

Muthaiah Shellaiah,[1] Ying-Chou Chen,[2] Turibius Simon,[3] Liang-Chen Li,[4] Kien Wen Sun, [1,2,4*] and Fu-Hsiang Ko[3]

[1] Department of Applied Chemistry, National Chiao Tung University, Hsinchu 300, Taiwan

[2] Department of Electronics Engineering, National Chiao Tung University, Hsinchu 300, Taiwan.

[3] Department of Materials Science and Engineering, National Chiao Tung University, Hsinchu 300, Taiwan.

[4] Center for Nano Science and Technology, National Chiao Tung University, Hsinchu 300, Taiwan.

*Correspondence: kwsun@mail.nctu.edu.tw

Table of contents:

Synthetic and data collection procedures (**S3-S5**)

Synthesis of **NDS1** and equilibrium states of S1 **(S6)**

FTIR spectra of p-ND, NDA, **NDS1** and S1 (**S7**)

Raman spectra of p-ND, NDA and **NDS1 (S8)**

DLS and Zeta potential of **NDS1** at 100 μg/mL dispersion in water **(S9)**

EDX, XPS and XRD of **NDS1 (S10-S11)**

SEM images of **NDS1** in presence of different metal ions **(S12-S13)**

SEM and TEM images of diamond nanowires (Cd^{2+}-**NDS1** NWs) **(S14)**

FTIR of **NDS1** in presence of Cd^{2+} ions **(S15)**

Schematic of feasible Cd^{2+} mediated self-assembly of **NDS1 (S15)**

EDX, XPS, AFM and XRD of **NDS1** in presence of Cd^{2+} ions **(S16-S19)**

Raman data of **NDS1** in presence of various metal ions **(S20-S21)**

Pad and alignment mark design by AutoCAD **(S22-S23)**

SEM of Cd^{2+}-**NDS1** NWs after Pt deposition **(S24)**

Interconnection diagram by AutoCAD and Single Cd^{2+}-**NDS1** NW with 4 Au contacts (**S25**)

Plots of electrical conductivity, resistance and static resistivity between 80-300 K **(S26-S27)**

Activation energy ($E_{a)}$ calculation from "lnR *Vs* 1/T" and lnσ *Vs* 1/T" plots **(S28)**

Plots of "Conductance *Vs* 1/T" and "lnR Vs $T^{(-1/4)}$" **(S29)**

MOSFET based conductivity measurements **(S30-S32)**

1. Synthesis of NDS1

The **NDS1** (Figure 1) was synthesized through the modified procedure as follows. 100 mg of commercially available nanodiamond powder (p-ND) with 40 ml of H_2SO_4:HNO_3 (9:1) was injected and refluxed for 12 hours. After the completion of reaction, it was filtered, washed several times with deionized (DI) water, and then dried under vacuum to provide the ND-Acid (NDA). The NDA was mixed with 100 mL of $SOCl_2$ and 2 mL DMF and refluxed for 12 hours under inert atmosphere. Next, the remaining brown liquid was decanted and then dried under N_2 flow to provide the ND-Acid chloride. To avoid the moisture sensitive reactions of ND-Acidchloride, it was consumed directly without any further purification. Towards the suspension of ND-Acidchloride in 100 mL of Toluene, excess of 4-Amino-5-phenyl-4H-1,2,4-triazole-3-thiol (**S1**) in 15 mL of Toluene was added and refluxed at 80^0 C for 12 hours to afford **NDS1**. Overall, afordable yield was achieved in all steps.

2. SEM and DLS data

To obtain SEM, EDX, Zeta potential, and DLS data of **NDS1**, 100 microgram (µg) of these derivatives were dispersed in 1 mL of water and utilized as such. **NDS1** nanowires (DNWs) with metal ions were first dispersed in 10 µg/mL solution and then drop-casted on Si-wafer and dried at 50 °C for 15 minutes to collect SEM and EDX data.

3. TEM and AFM studies

For TEM and AFM analysis 1 ng of **NDS1** was dispersed in 1 mL. However, for HR-TEM invetigation of **NDS1** agglomeration, 100 ng in 1 mL water was consumed. Likewise, for TEM images of Cd^{2+}-**NDS1** NWs, 10 µg of **NDS1** mixed with 10 micromole (µM) of Cd^{2+} ions was diluted to the ratio of 1 ng/1 nM in 1mL of water. AFM image of Cd^{2+}-**NDS1** NWs assembly was obtained by drop-casting over the Si-

wafer substrate. For the TEM investigations, **NDS1** with or without Cd^{2+} ions were dispersed over copper-carbon grid.

4. FTIR, Raman and XPS spectra

For FTIR, Raman, and XPS analyses, the samples (100 μg/mL) were drop-casted on Si wafers and then annealed at 60 °C for 30 min. We used a clean Si wafer as a background reference for both spectroscopic analyses. During Raman interrogations, each measured spectrum was obtained from averaging over 30 collected data, wherein five different locations on each sample were considered from six samples. The reproducibility of the presented data is higher than 85%.

5. Metal ion induced G-DNWs formation

For this data collection, all the metal ions (Na^{+}, Ni^{2+}, Fe^{3+}, Cd^{2+}, Ca^{2+}, Ga^{3+}, Cr^{3+}, Cu^{2+}, Fe^{2+}, Mg^{2+}, Au^{3+}, Y^{3+}, and Al^{3+}) were dissolved in water medium at 1×10^{-2} M concentration of their respective chloro- and perchlorate compounds. Similarly, Ag^{+}, Co^{2+}, Zn^{2+}, Pb^{2+}, Mn^{2+}, and Hg^{2+} metal cations were dissolved in water medium at 1×10^{-2} M concentration of their respective acetate salts. From the above stock solutions, 100 μM of each metal ion was added to 100 μg/mL dispersion of **NDS1** and incubated for 45 minutes to demonstrate the nanowire like assemblies. Each mixture was directly subjected to SEM analysis to determine the reproducibility from 100 collected data. The percentage reproducibility was calculated as follows. Under similar condition, each metal ion was incubated with **NDS1** dispersion in 20 vials for 45 minutes. Thereafter, these dispersive mixtures were drop-casted over the well cleaned Silicon wafers and were subjected to SEM investigations. During the SEM interrogations, the existence of G-DNWs with metal ions on Si-wafers was examined at five different locations and the overall results were taken into account to calculate the reproduciblity. The reproducibility of Cd^{2+}-**NDS1** NWs seems to be higher than that of other ions due to the effective formation of sp^2 graphite layer over the DNWs along with impurity channels. Hence, the above procedure was continued to

generate less aggregated and scattered Cd^{2+}-**NDS1** NWs formation with dispersed dilution to 100 ng in 1 mL of DI-water. For TEM studies, the above homogeneous dispersion was further reduced to 1 ng/mL in DI-water.

6. Stability of Cd^{2+}-NDS1 NWs

The above wet synthesized Cd^{2+}-**NDS1** NWs were initially formed at low dispersion 100 μg of **NDS1** and 100 μM of Cd^{2+} ions in 1 mL of water. However, further dispersion by adding more DI-water resulted to the breaking of longer NWs (~50 μm) into shorter ones (a few microns). These short Cd^{2+}-**NDS1** NWs were intended to form agglomerated particles. If the as-grown Cd^{2+}-**NDS1** NWs were mixed with other metal ions, they were also intended to form agglomerated **NDS1** nanoparticles or small nanorods. Note that the fabricated Cd^{2+}-**NDS1** NWs were found to be highly stable during the ultrasonication with DI-water and iso-propyl alcohol (IPA).

7. XRD, Raman, and FTIR Interrogations on Cd^{2+}-NDS1 NWs

The XRD of **NDS1** was done by means of powder XRD analysis. The Cd^{2+}-**NDS1** NWs powder were obtained by stirring the **NDS1** powder with Cd^{2+} ions for 45 minutes and centrifuged and washed with DI-water to completely remove the excess Cd^{2+} ions. The final product was then dried in oven under vaccum. These dried Cd^{2+}-**NDS1** NWs powder was then subjected to XRD analysis. The dried Cd^{2+}-**NDS1** NWs powder was first dispersed in water, and then drop-casted on Si-wafer to be interrogated using FTIR. Following the aforementioned approaches, all the metal ions were engaged to produce the coresponding G-DNWs powders, which were then dispersed in water and drop-casted over the cleaned Si-wafer and then subjected to Raman studies.

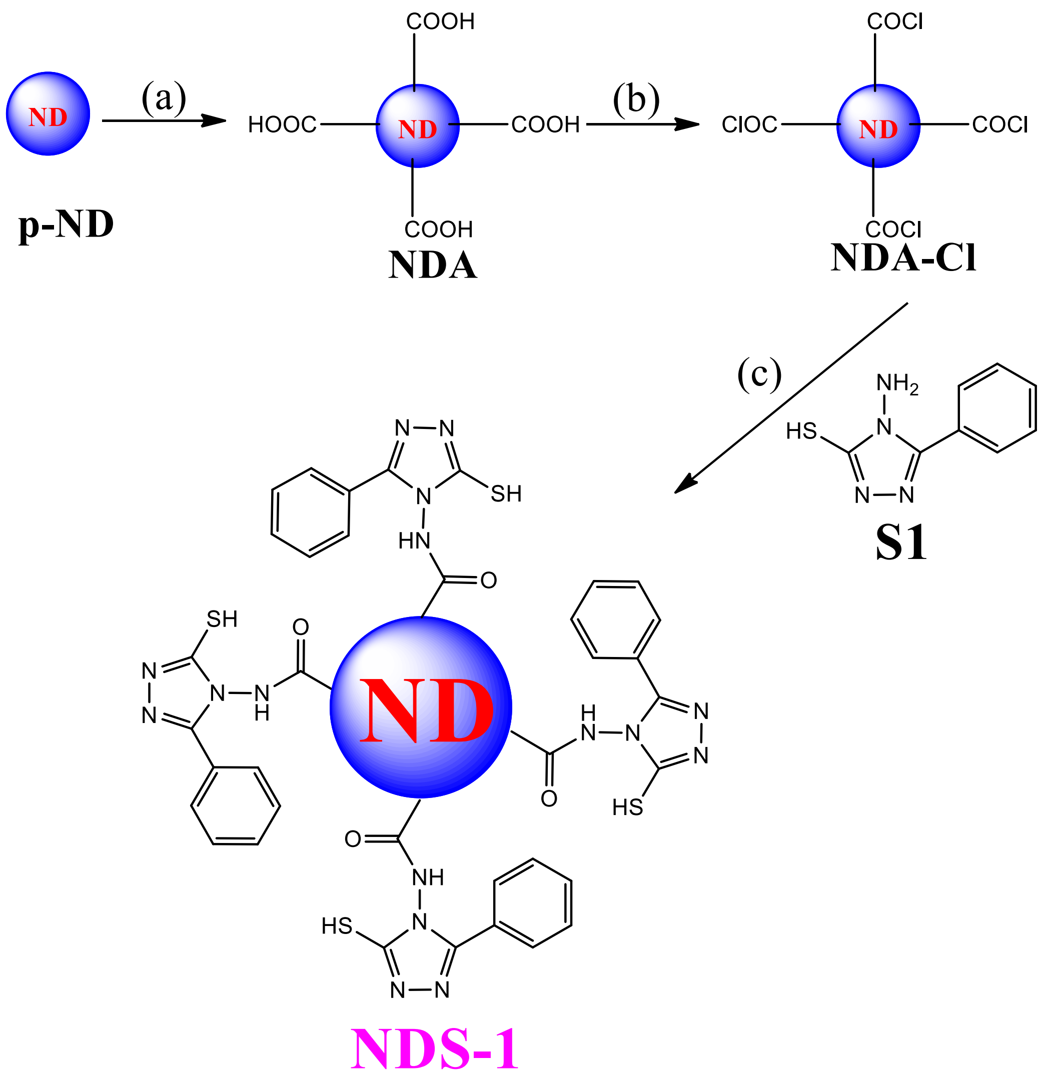

Figure S1. Synthesis of **NDS1** (a) H_2SO_4:HNO_3 (9:1), reflux for 12 h; (b) $SOCl_2$:DMF (10:0.2), reflux for 12 h; (c) 4-Amino-5-phenyl-4H-1,2,4-triazole-3-thiol (**S1**), Toluene, reflux at 100 °C for 12 h.

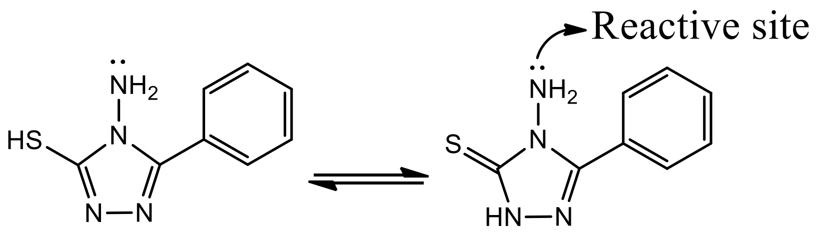

S1 at Equilibrium

Figure S2. Equilibrium states of S1, which avoids the competitive reaction with thiol or secondary amine groups.

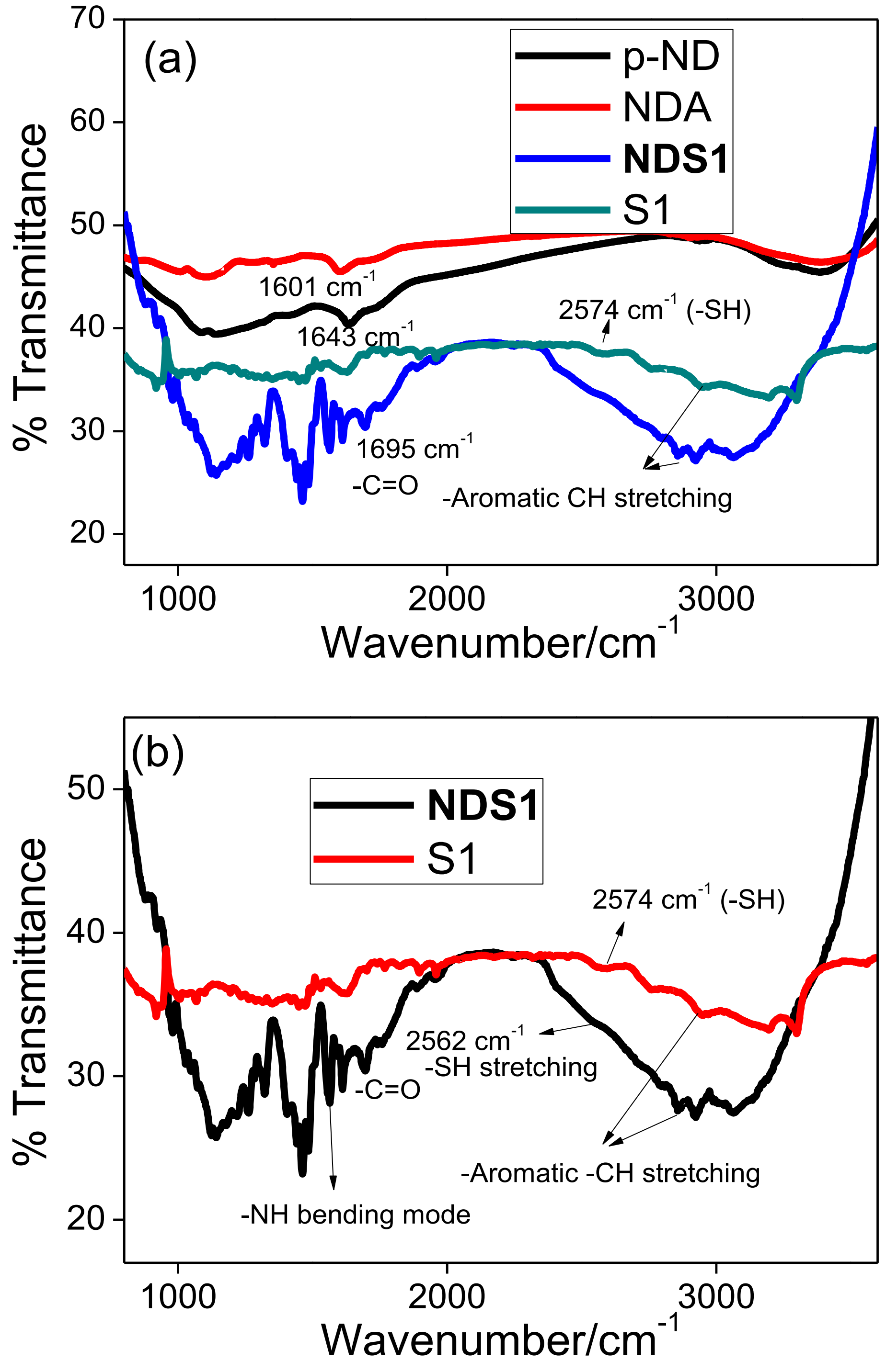

Figure S3. FTIR spectra of (a) p-ND, NDA, **NDS1,** and S1 and (b) **NDS1** and S1.

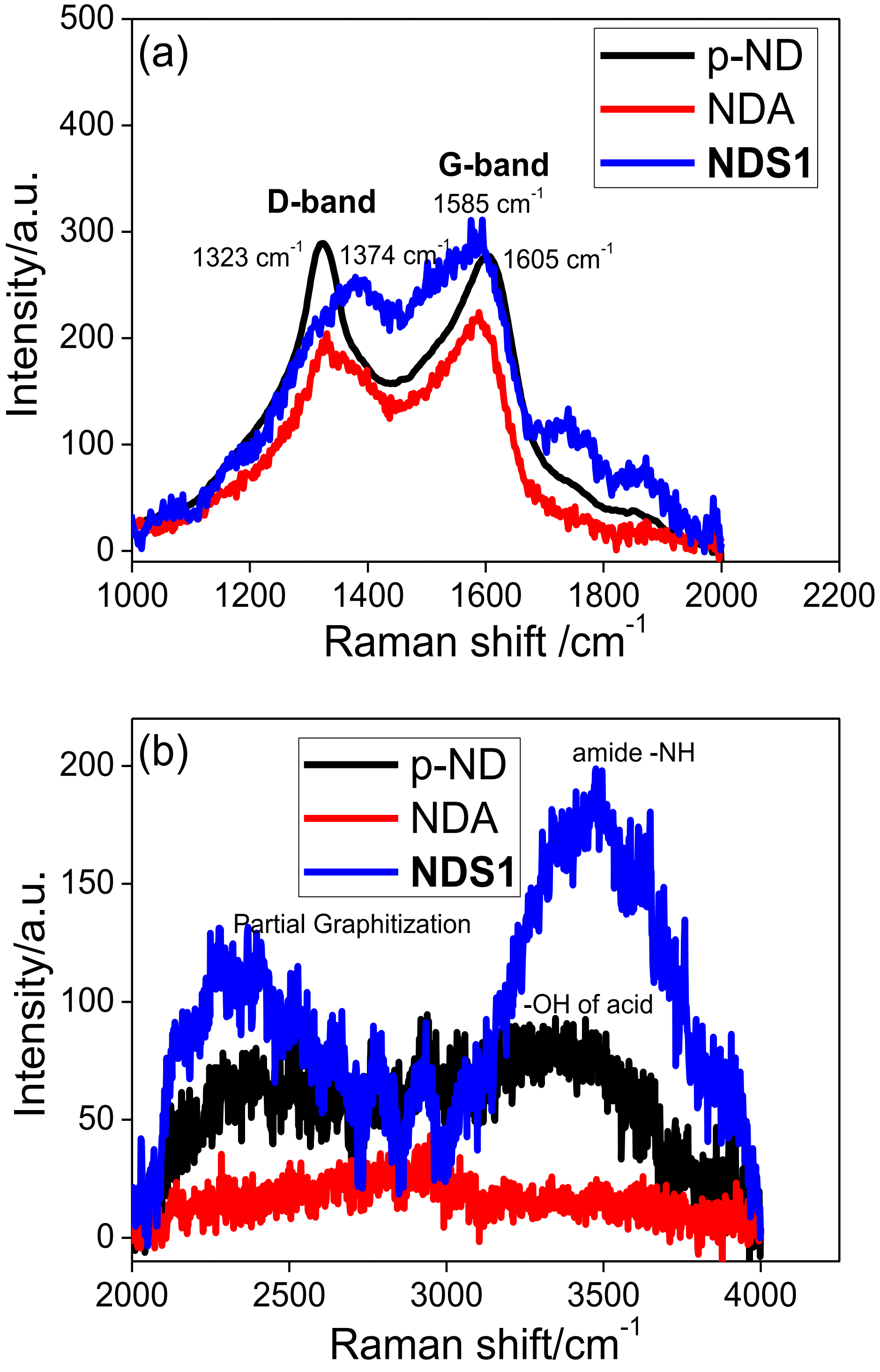

Figure S4. Raman spectra of p-ND, NDA, and NDS1 from (a) 1000 - 2200cm^{-1} (b) 2000 - 4000 cm^{-1}.

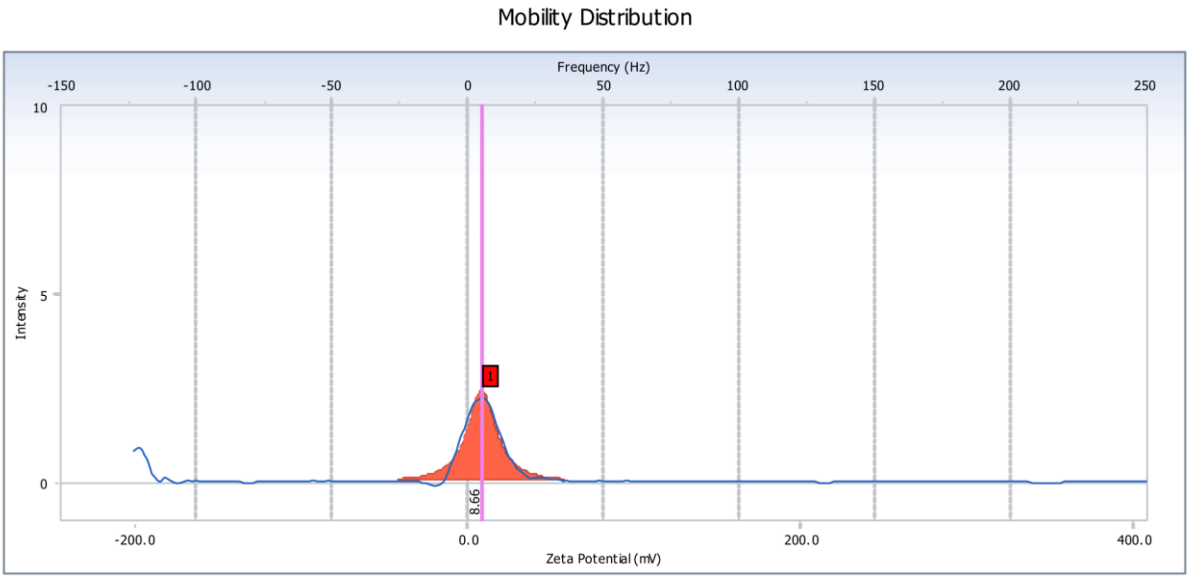

Figure S5. Zeta potential of **NDS1** at 100 μg/mL dispersion in water.

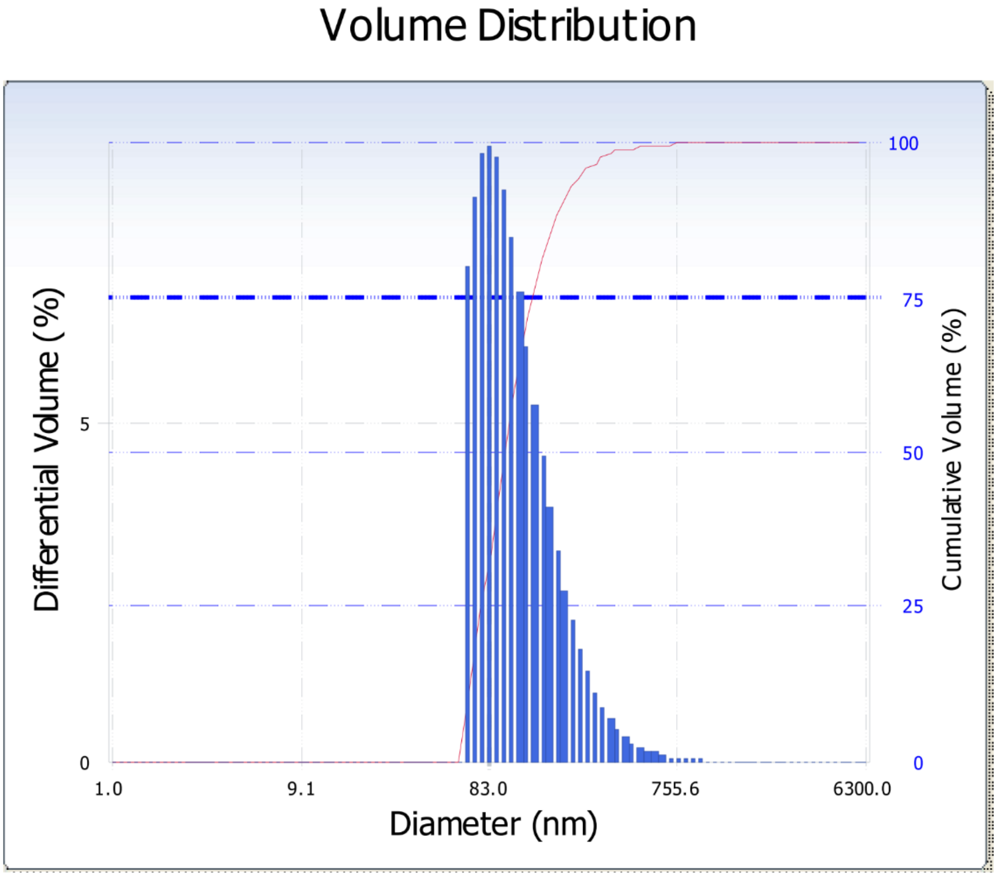

Figure S6. DLS of **NDS1** at 100 μg/mL dispersion in water.

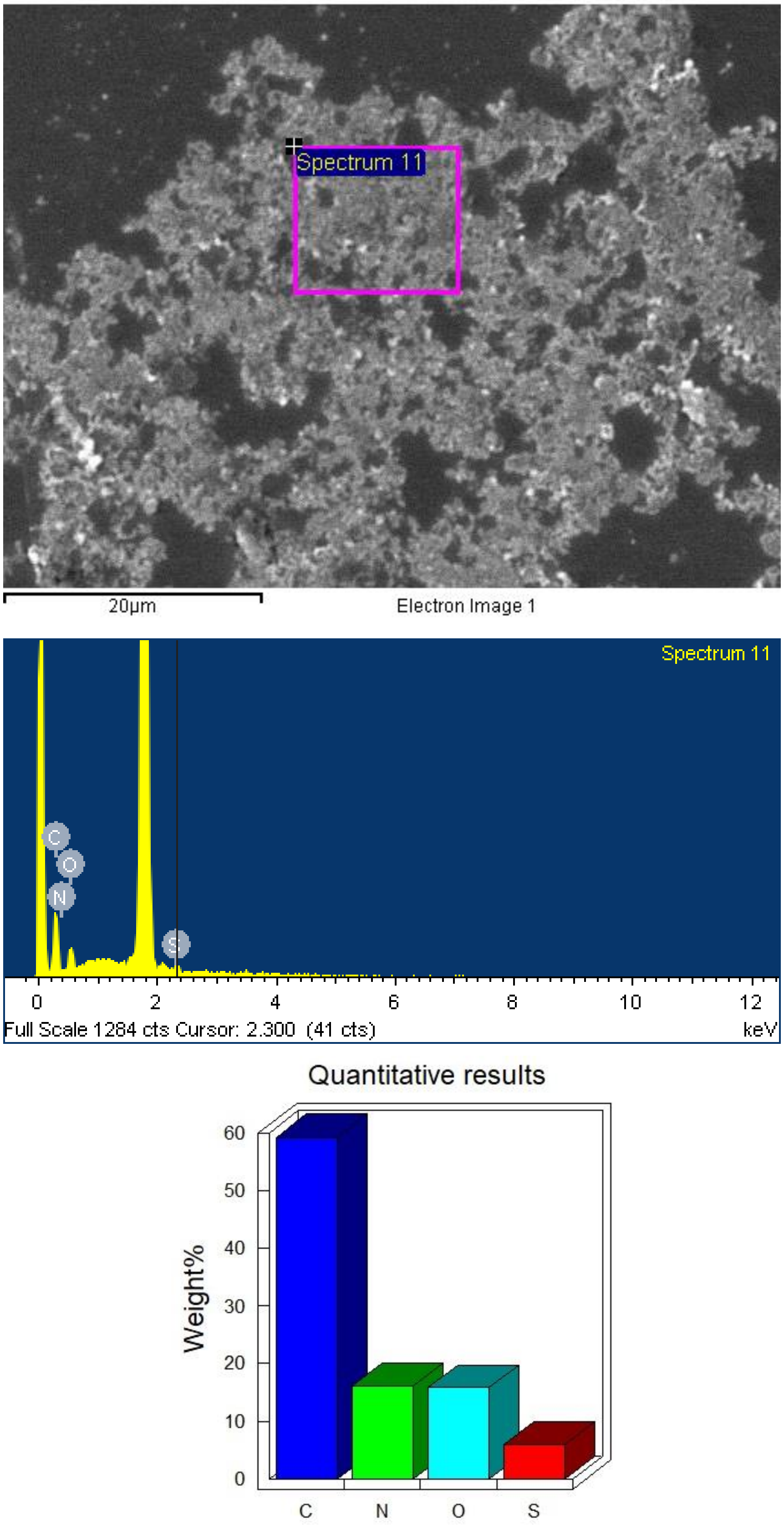

Figure S7. EDX spectrum representing the presence of C, N, O and S atoms in **NDS1**.

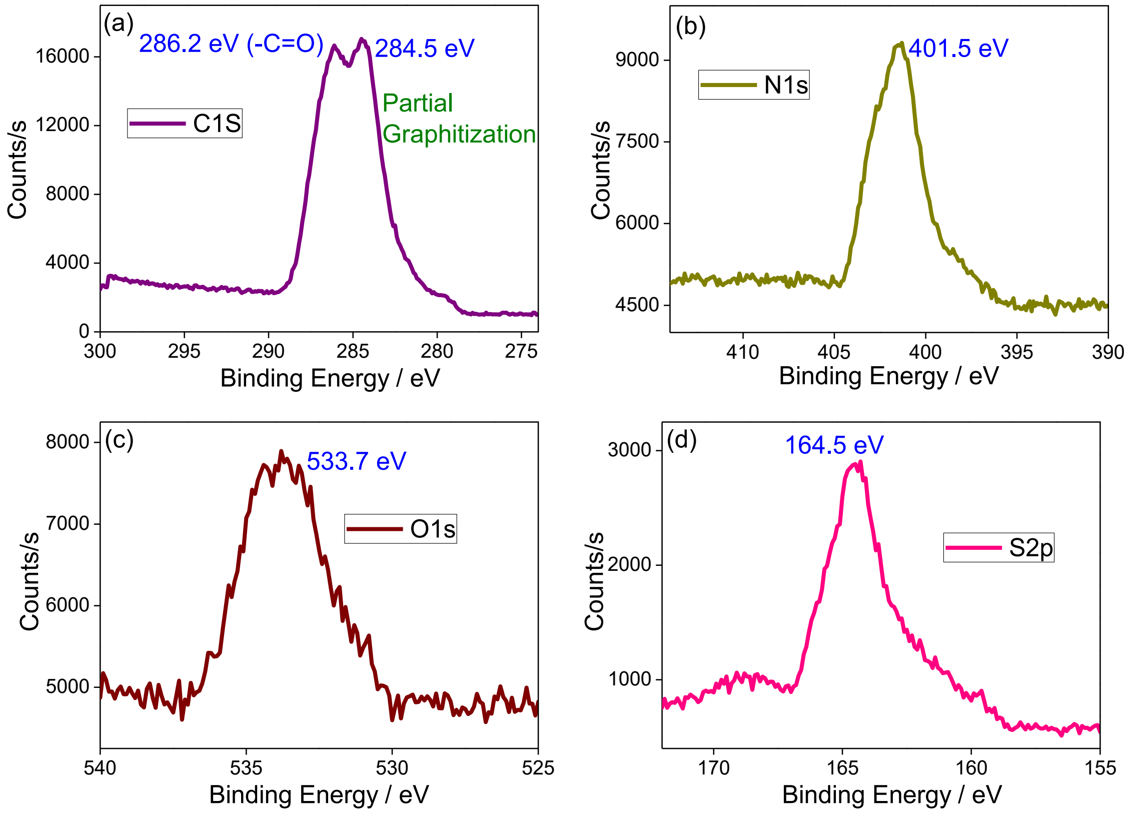

FigureS8. XPS spectra representing (a) C1s, (b) N1s, (c) O1s and (d) S2p peaks of **NDS1**.

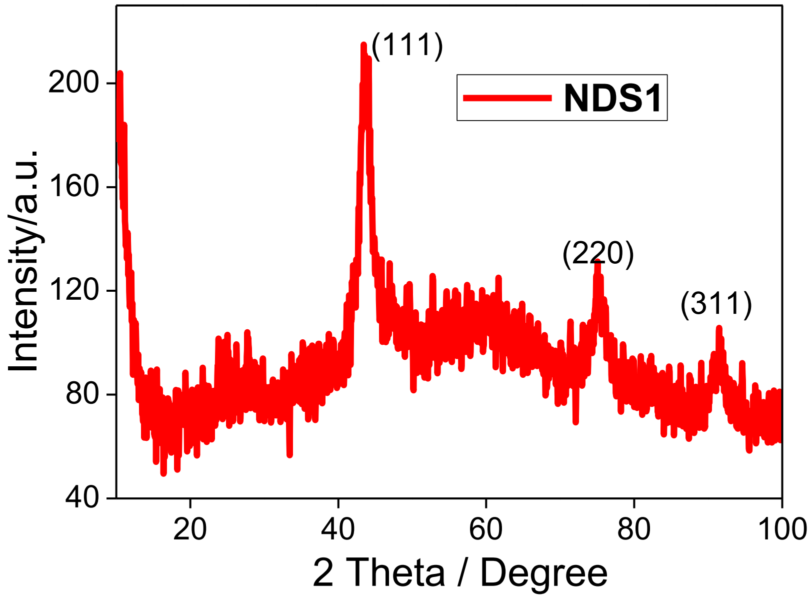

Figure S9. XRD spectrum of **NDS1** representing (111), (220) and (311) patterns of nanodiamond.

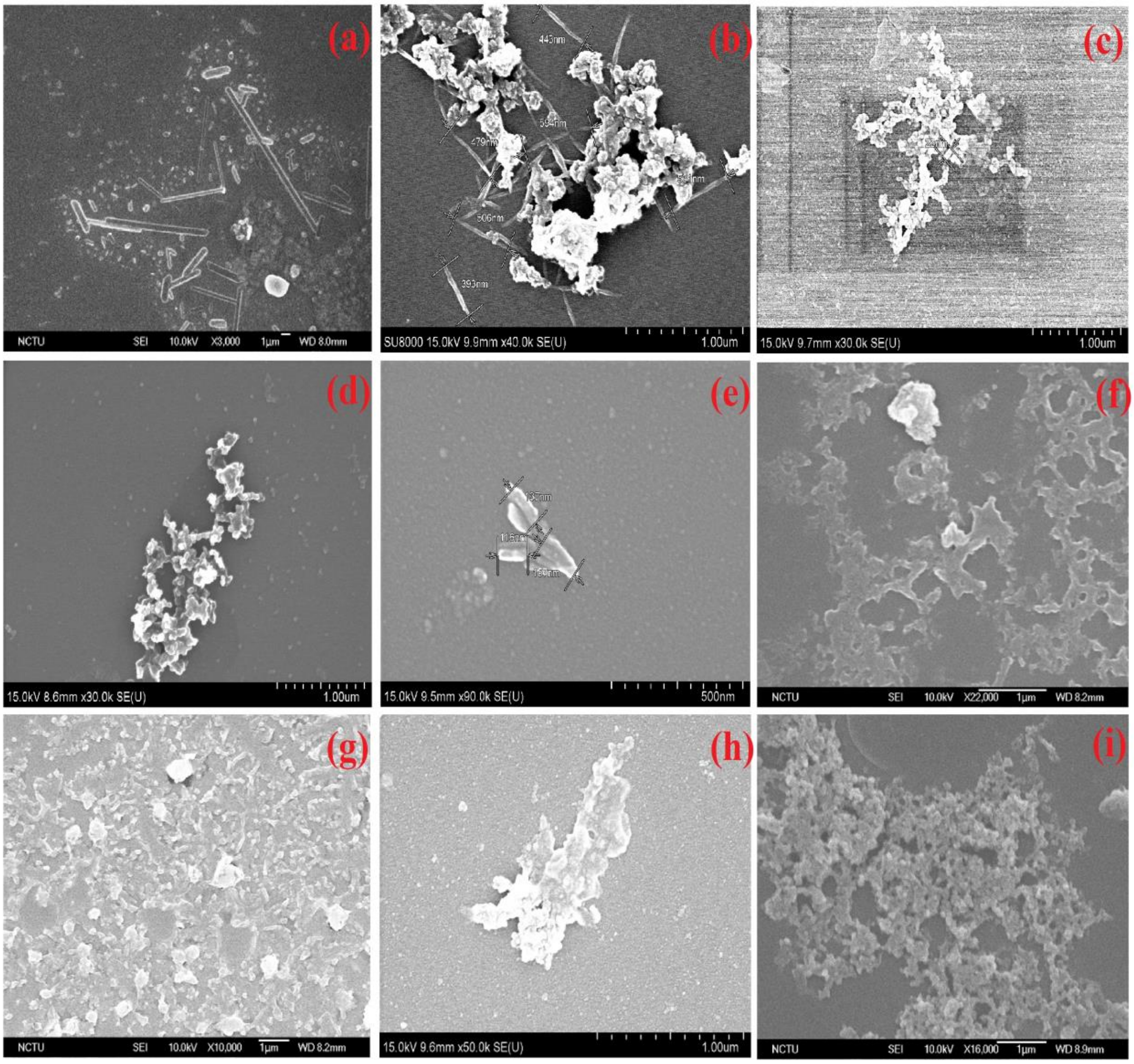

Figure S10. SEM images of **NDS1** in the presence of (a) Cd^{2+}, (b) Ag^{+}, (c) Fe^{3+}, (d) Hg^{2+}, (e) Pb^{2+}, (f) Al^{3+}, (g) Cr^{3+}, (h) Co^{2+} and (i) Mn^{2+} ions.

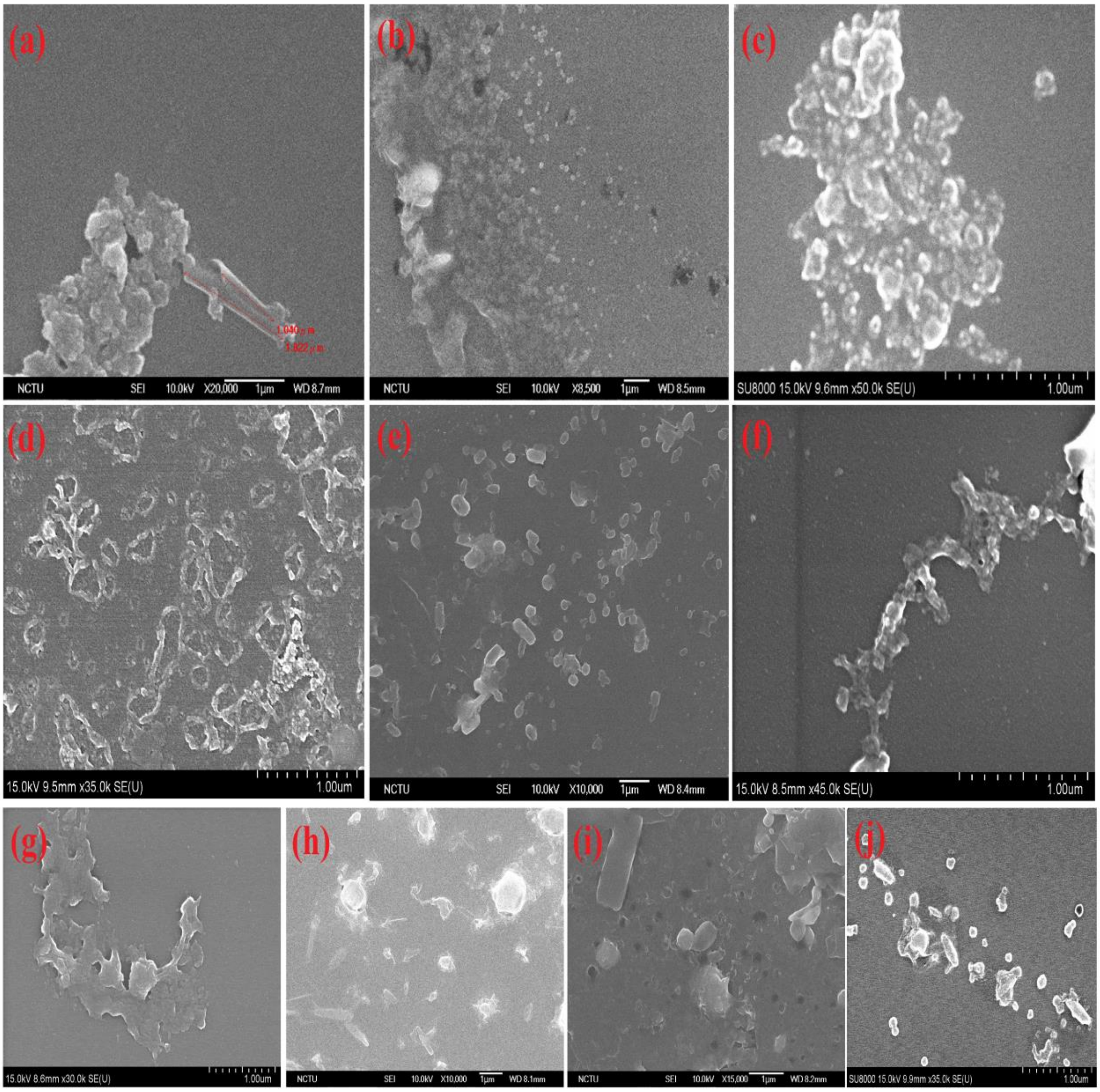

Figure S11. SEM images of **NDS1** in the presence of (a) Ni^{2+}, (b) Au^{3+}, (c) Ga^{3+}, (d) Cu^{2+}, (e) Fe^{2+}, (f) Zn^{2+}, (g) Mg^{2+}, (h) Ca^{2+}, (i) Y^{3+} and (j) Na^{+} ions.

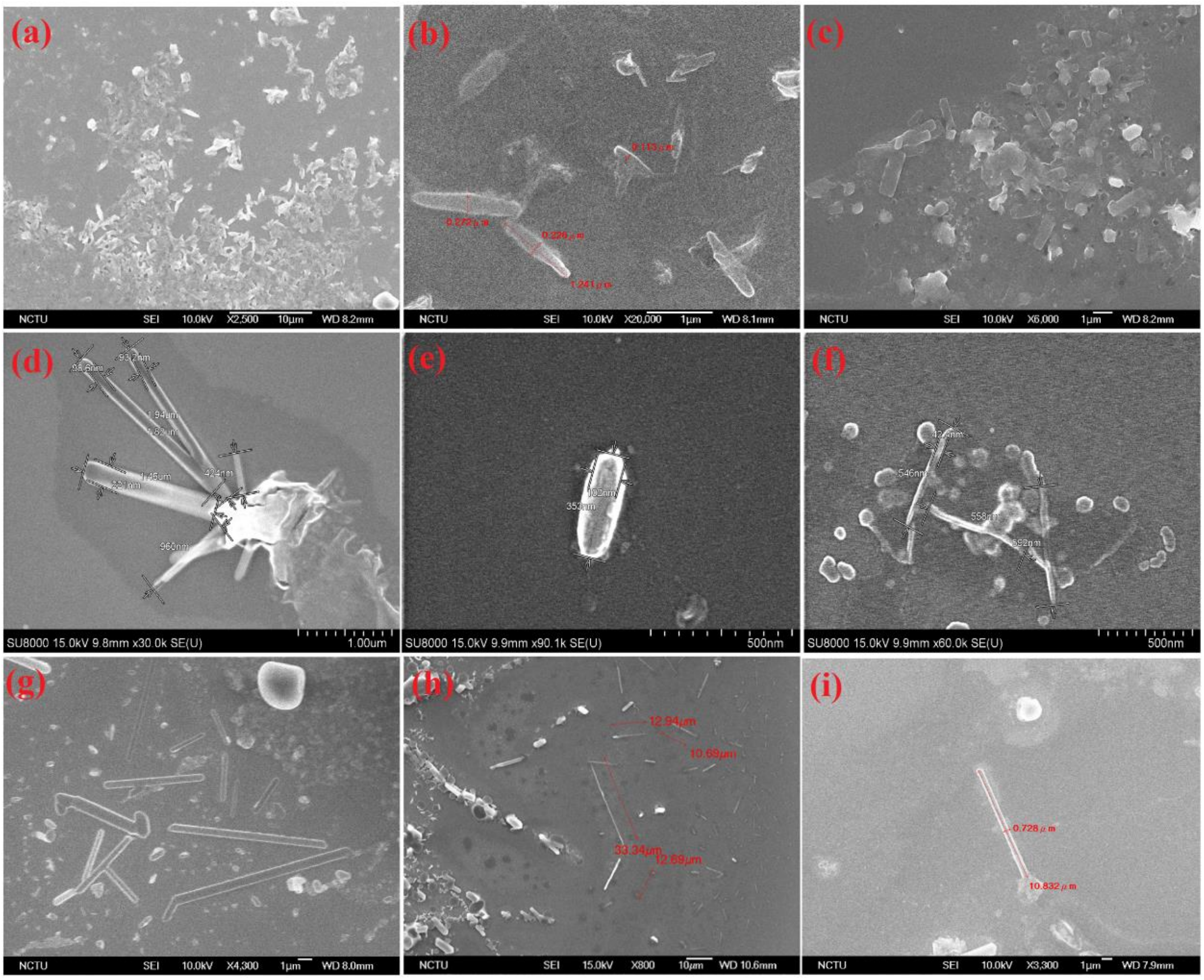

Figure S12. SEM images of **NDS1** in the presence of Cd^{2+} ions (10 µg /mL in water) and scanned at different regions (a)-(i).

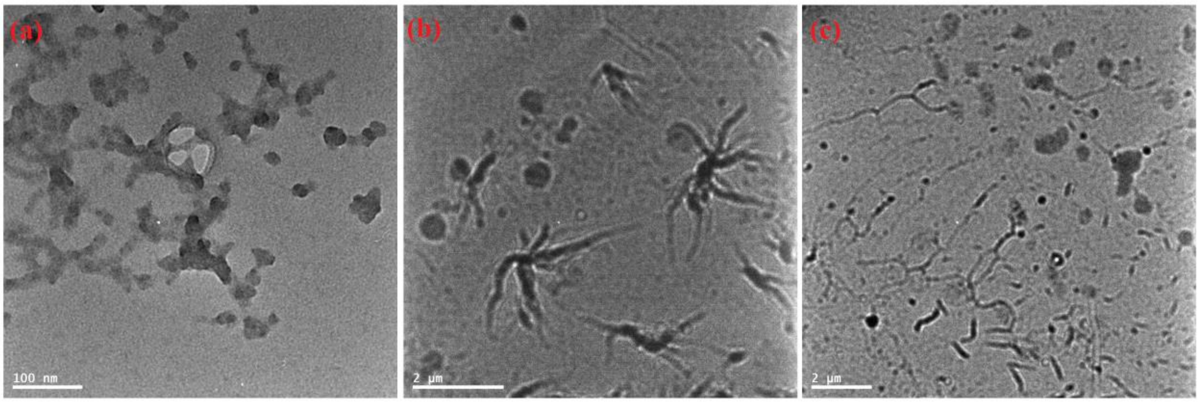

Figure S13. TEM images of **NDS1** in the presence of Cd^{2+} ions (1 ng /mL in water) and scanned at different regions (a)-(c).

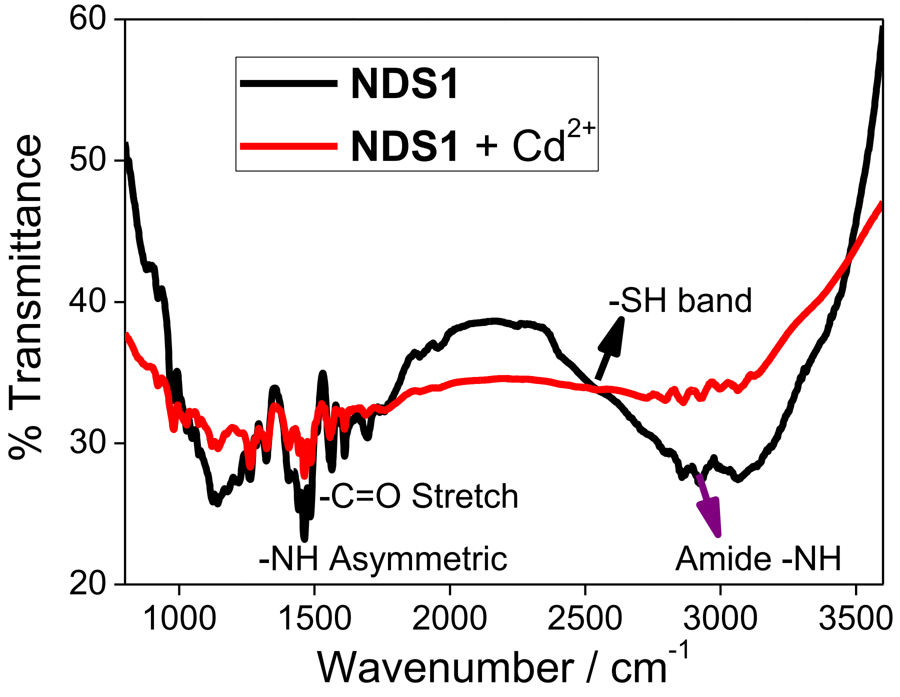

Figure S14. FTIR spectra of **NDS1** and **NDS1** + Cd^{2+} ions.

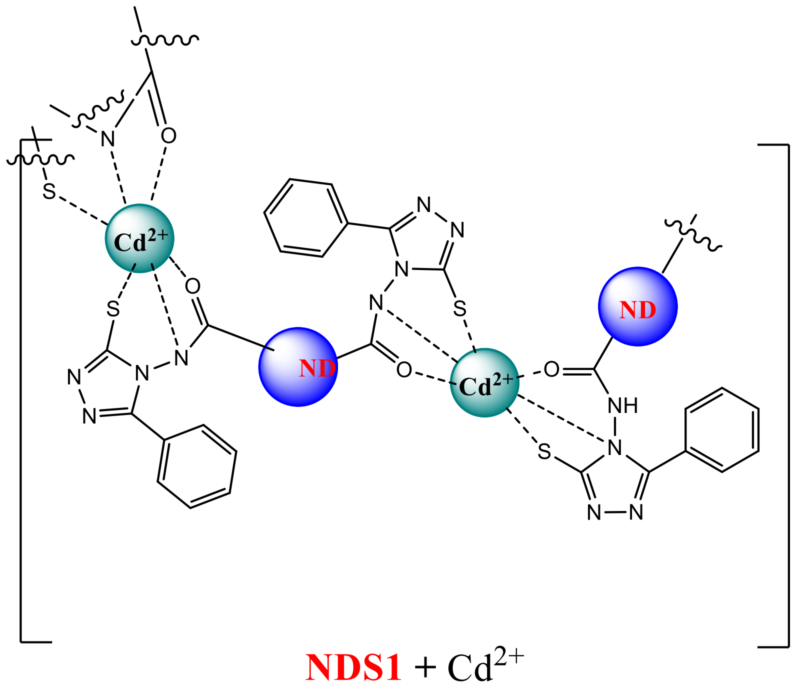

Figure S15. Schematic representation of feasible repeating units present in Cd^{2+} ions mediated self-assembly of **NDS1** in NW formation.

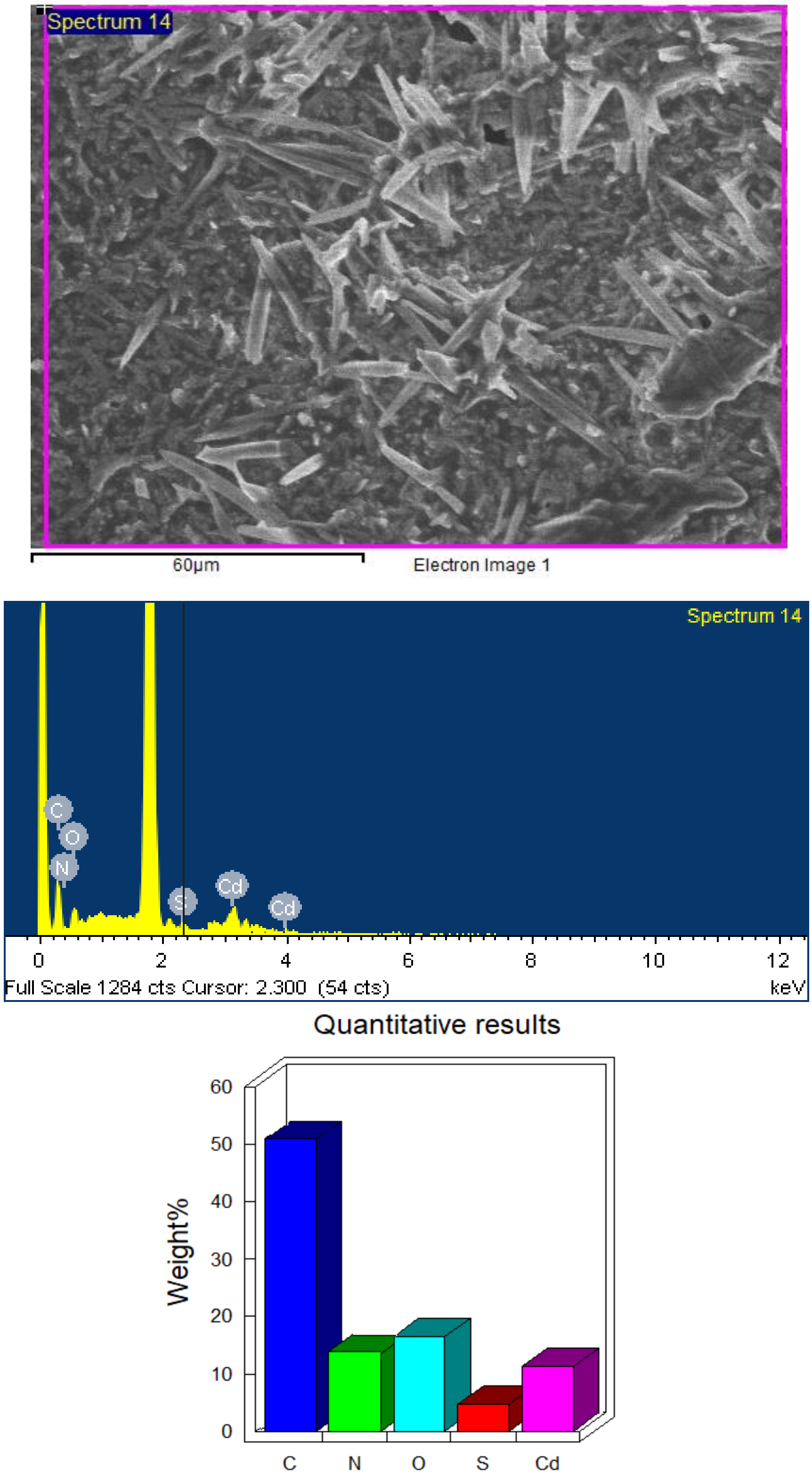

Figure S16. EDX spectrum of Cd^{2+}-**NDS1** NWs representing the presence of C, N, O and S and Cd^{2+} atoms.

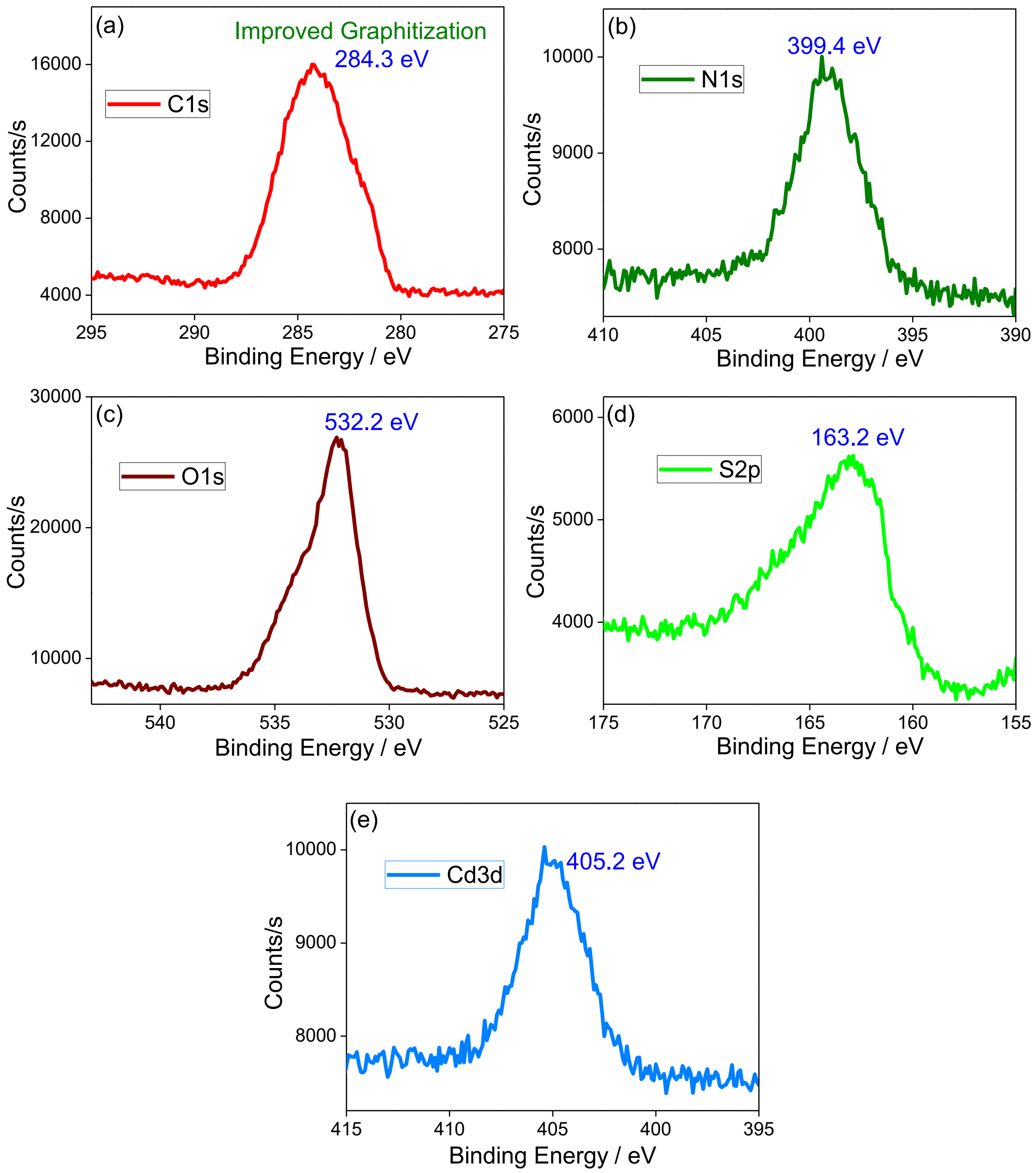

Figure S17. XPS spectra of Cd^{2+}-**NDS1** NWs representing peaks of (a) C1s, (b) N1s, (c) O1s, (d) S2p and (e) Cd3d.

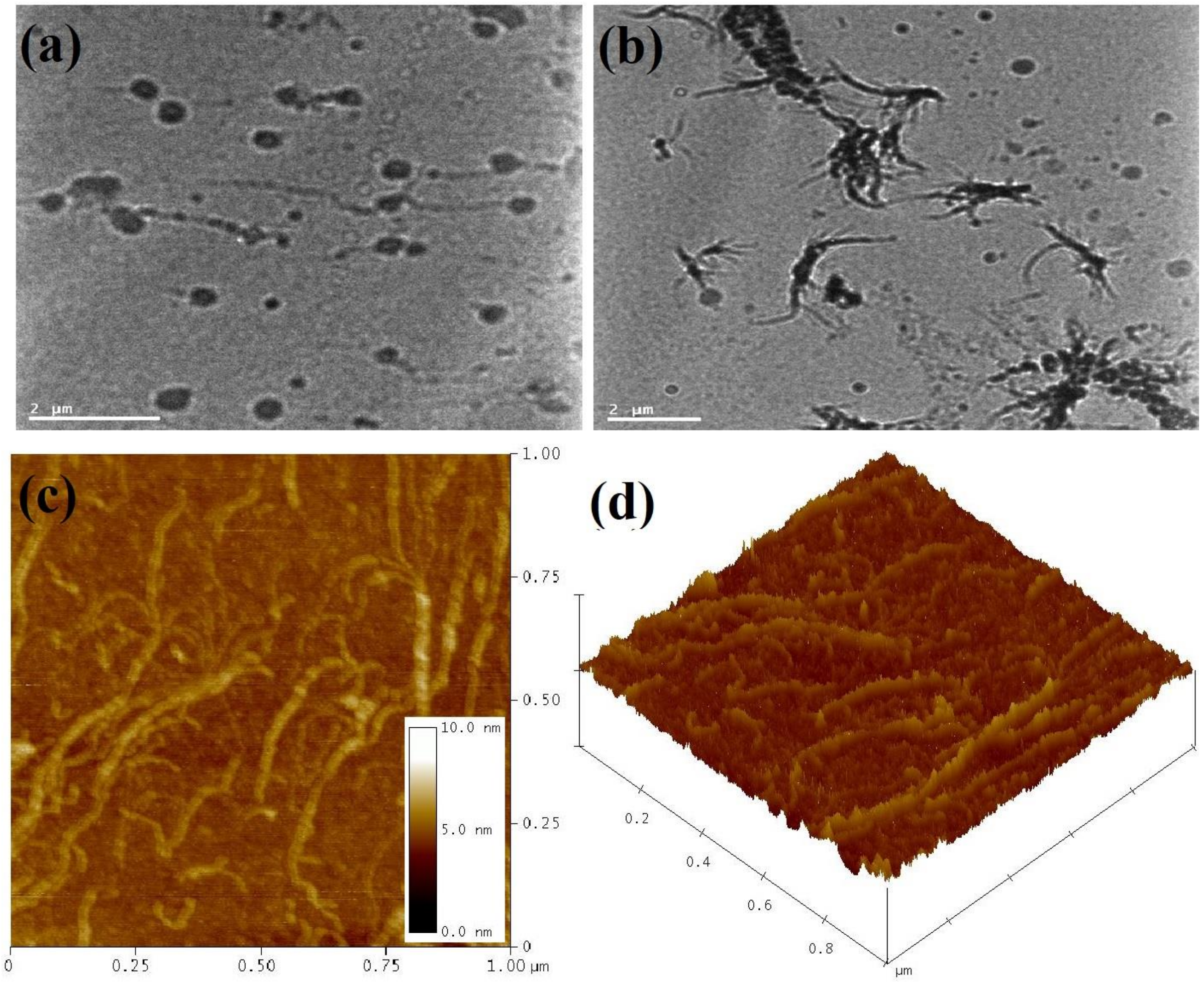

Figure S18. (a) and (b) TEM images representing the self-assembly of **NDS1** nanoparticles in the presence of Cd^{2+} ions towards the formation of nanowires (NWs), (c) AFM images show the self-assembled **NDS1** with Cd^{2+} ions in the formation of NWs and (d) AFM top-view image of self-assembly of **NDS1** with Cd^{2+} in the formation of NWs.

Table S1. XPS data of **NDS1** and **NDS1** in the presence of Cd^{2+} ions.

Composition	**NDS1**	**NDS1** + Cd^{2+}
C1s	284.5 & 286.2 eV	284.3 eV
N1s	401.5 eV	399.4 eV
O1s	533.7 eV	532.2 eV
S2p	164.5 eV	163.2 eV
Cd3d	NA	405.2 eV

NA = Not Applicable

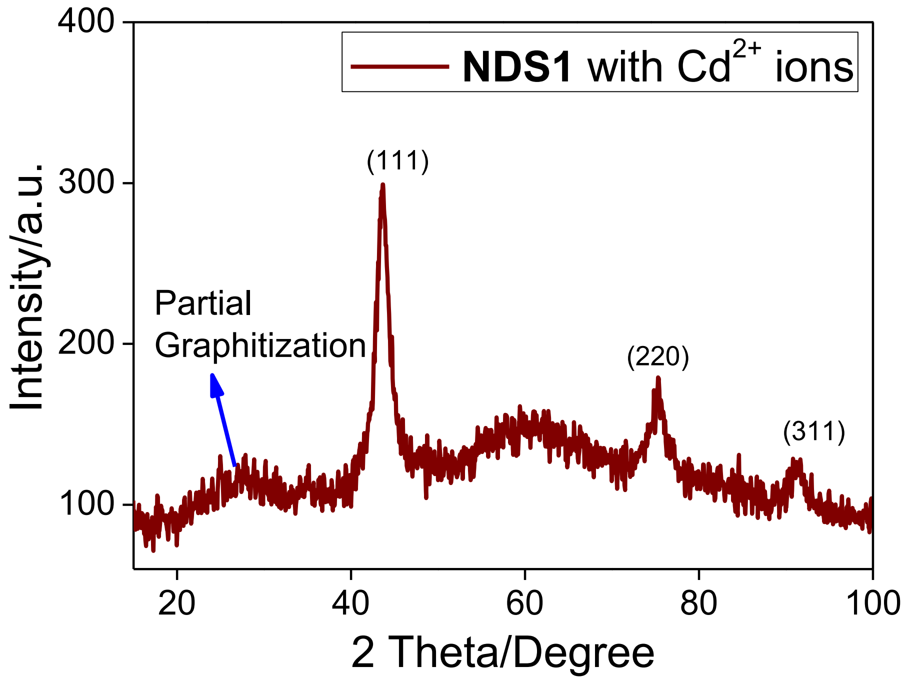

Figure S19. XRD spectra of **NDS1** with Cd^{2+} ions representing (111), (220) and (311) patterns of nanodiamond along with partial graphitization (002) at 25.5°.

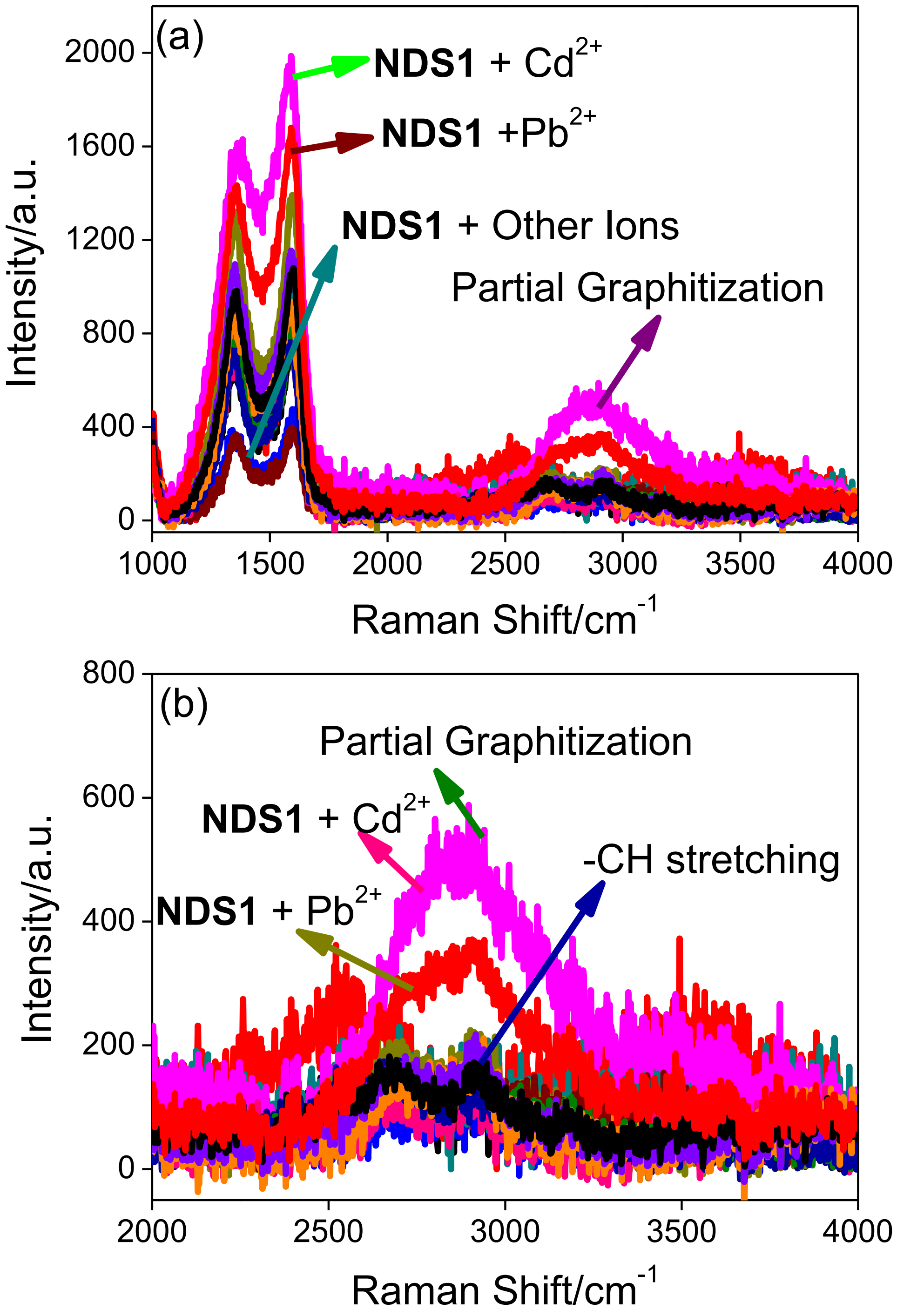

Figure S20. (a,b) Raman spectra of **NDS1** in the presence of metal ions from (a) 1000 - 4000 cm^{-1}(b) 2000 - 4000 cm^{-1}.

Table S2. D and G bands and I_G/I_D of **NDS1** in the presence of different metal ions

System	D-Band (cm^{-1})	Intensity (A.U.)	G-Band (cm^{-1})	Intensity (A.U.)	I_G/I_D (n = 30)
**NDS1** + Al^{3+}	1357	654	1605	753	1.15
**NDS1** + Co^{2+}	1347	881	1604	1024	1.16
**NDS1** + Cr^{3+}	1348	395	1605	457	1.16
**NDS1** + Fe^{3+}	1357	762	1604	919	1.21
**NDS1** + Cd^{2+}	1381	1577	1585	1998	1.27
**NDS1** + Fe^{2+}	1349	1308	1596	1383	1.06
**NDS1** + Ca^{2+}	1348	707	1595	795	1.12
**NDS1** + Ag^{+}	1358	316	1598	388	1.23
**NDS1** + Au^{3+}	1367	680	1596	780	1.15
**NDS1** + Zn^{2+}	1348	391	1605	460	1.18
**NDS1** + Mn^{2+}	1348	780	1596	826	1.06
**NDS1** + Na^{+}	1348	711	1586	758	1.07
**NDS1** + Hg^{2+}	1334	720	1602	790	1.10
**NDS1** + Ga^{3+}	1348	855	1586	926	1.08
**NDS1** + Mg^{2+}	1348	975	1605	1073	1.10
**NDS1** + Y^{3+}	1358	1080	1590	1160	1.04
**NDS1** + Pb^{2+}	1357	894	1595	1078	1.21
**NDS1** + Ni^{2+}	1358	1424	1596	1656	1.16
**NDS1** + Cu^{2+}	1348	765	1596	830	1.08

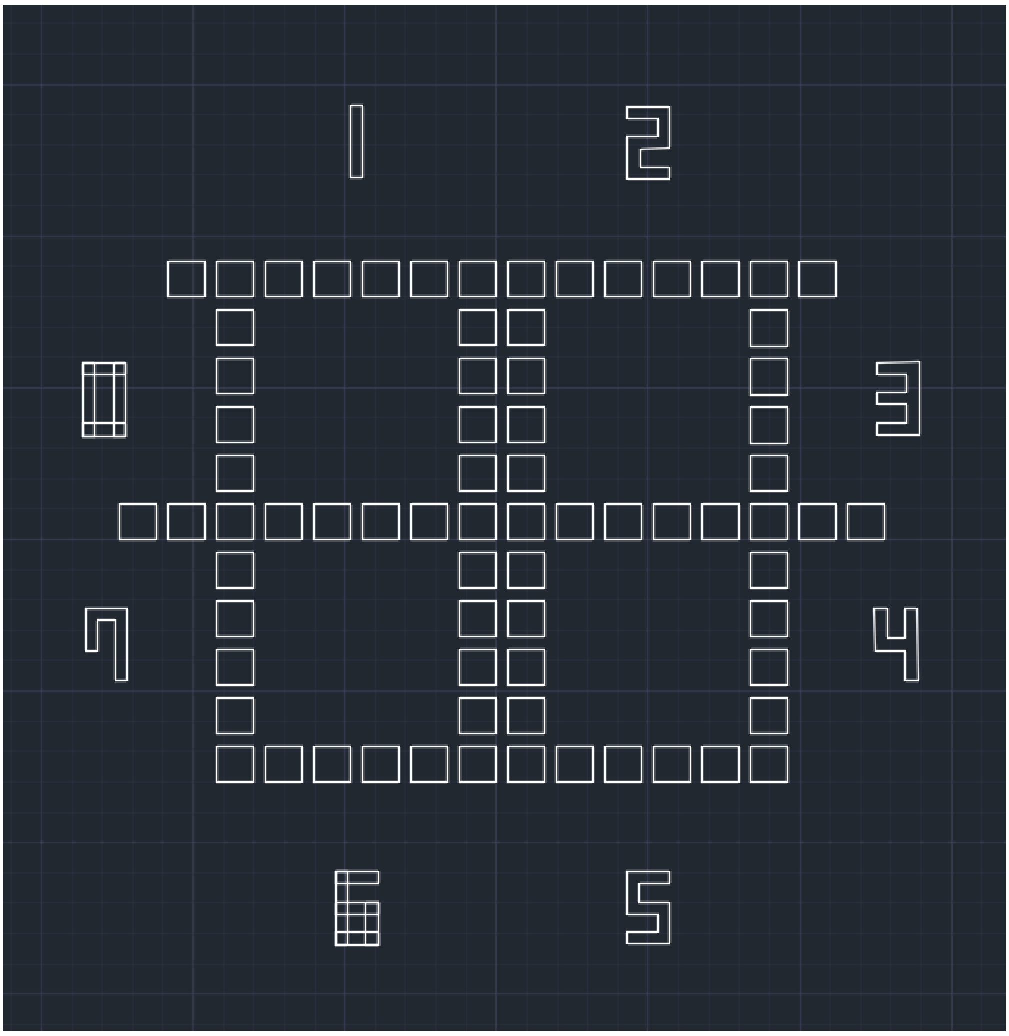

Figure S21. Pads design diagram by AutoCAD.

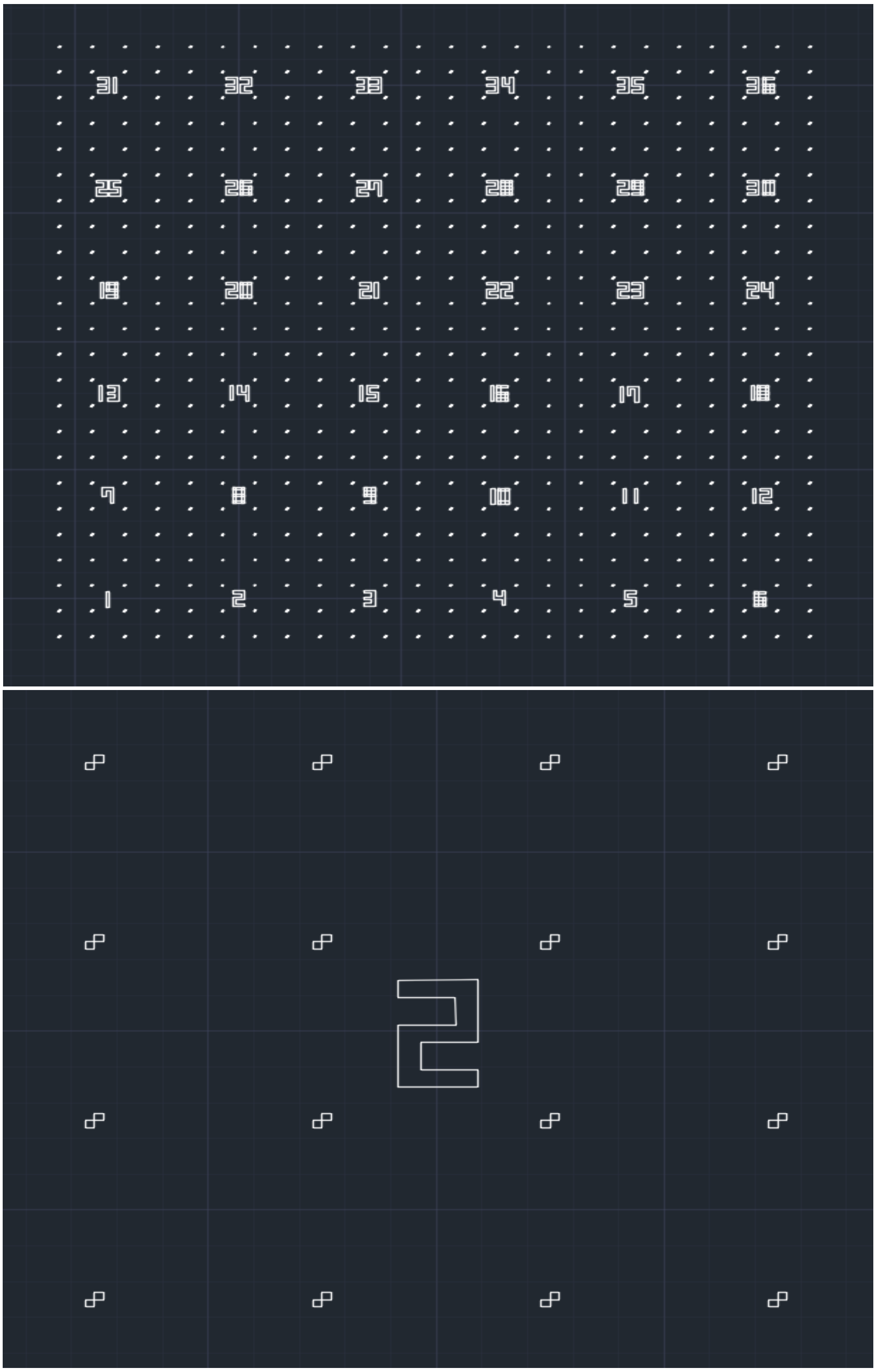

Figure S22. Alignment marks design diagram by AutoCAD.

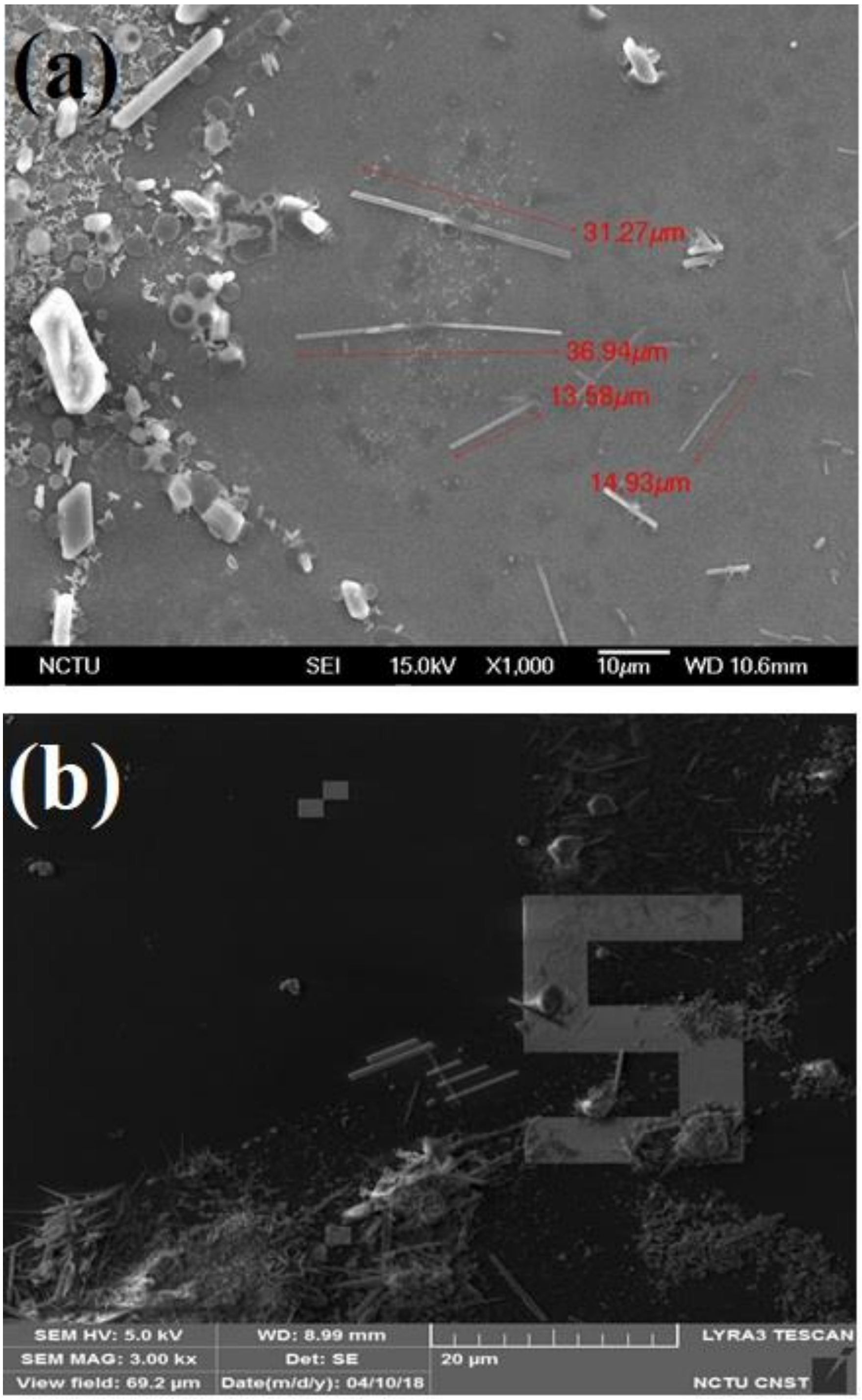

Figure S23. SEM images of (a) Cd^{2+}-**NDS1** NWs and (b) Cd^{2+}-**NDS1** NWs after Pt deposition by FIB to provide 4 contact single nanowire.

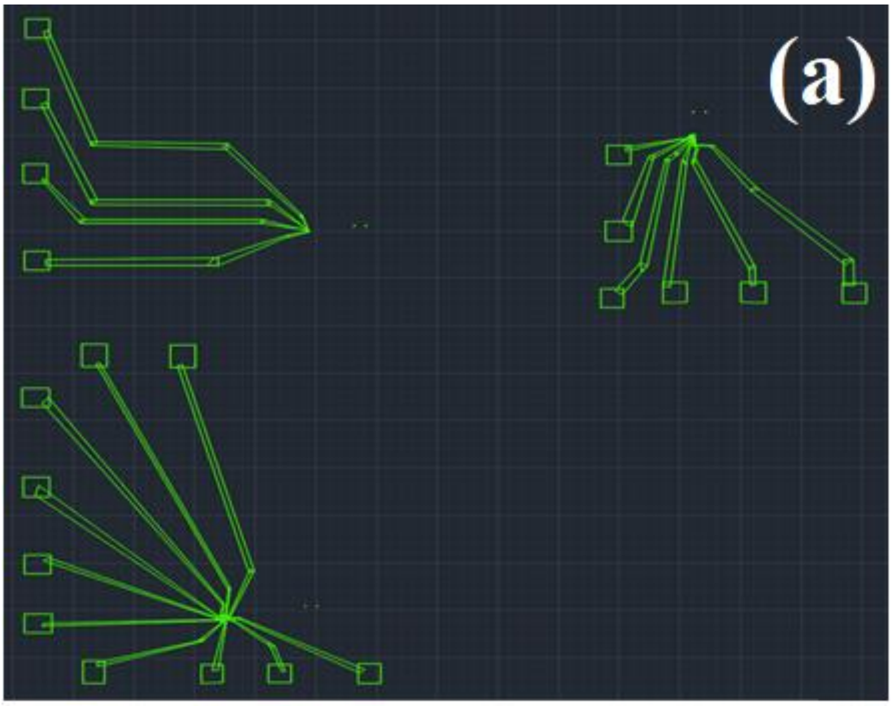

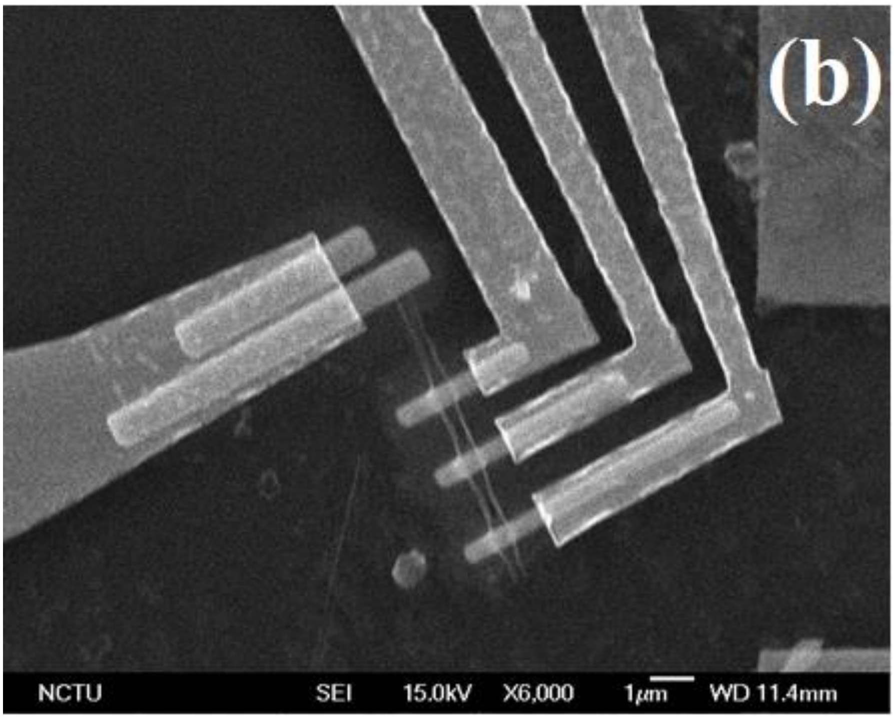

Figure S24. (a) Diagram of interconnections by AutoCAD and (b) Single Cd^{2+}-**NDS1** NW with 4 Au contacts after interconnecting.

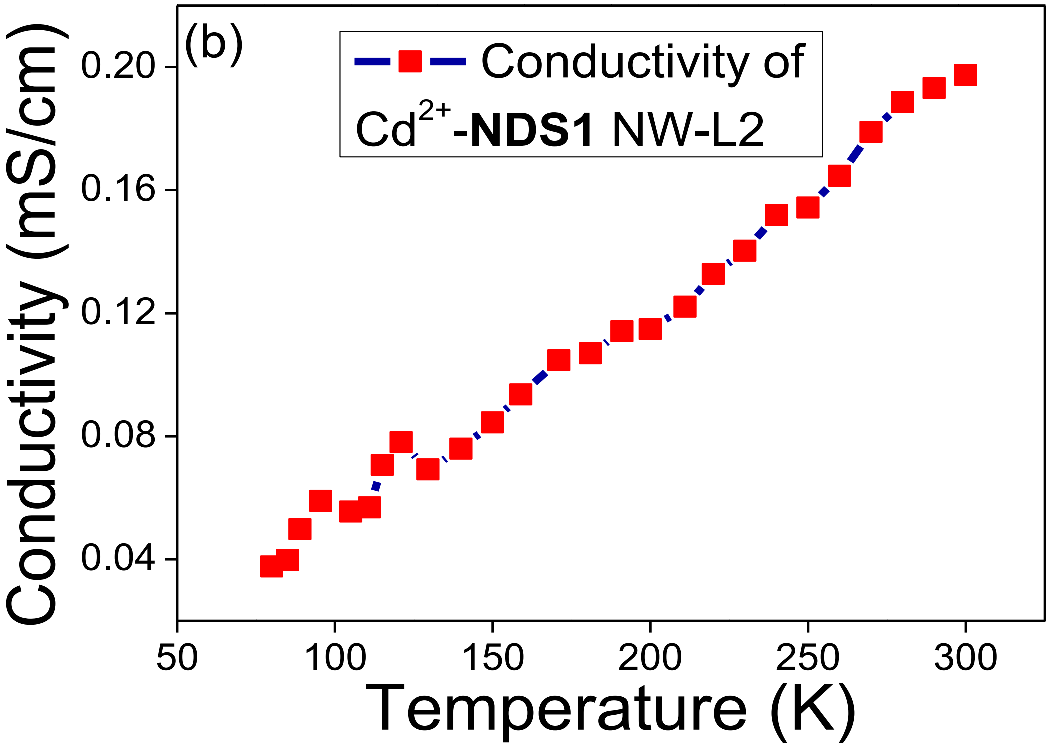

Figure S25. Temperature dependent conductivity of Cd^{2+}-**NDS1** NW- L2 [2-contacts, 2-point probe in vaccum (10^{-2} torr)] between 80 ~ 300 k.

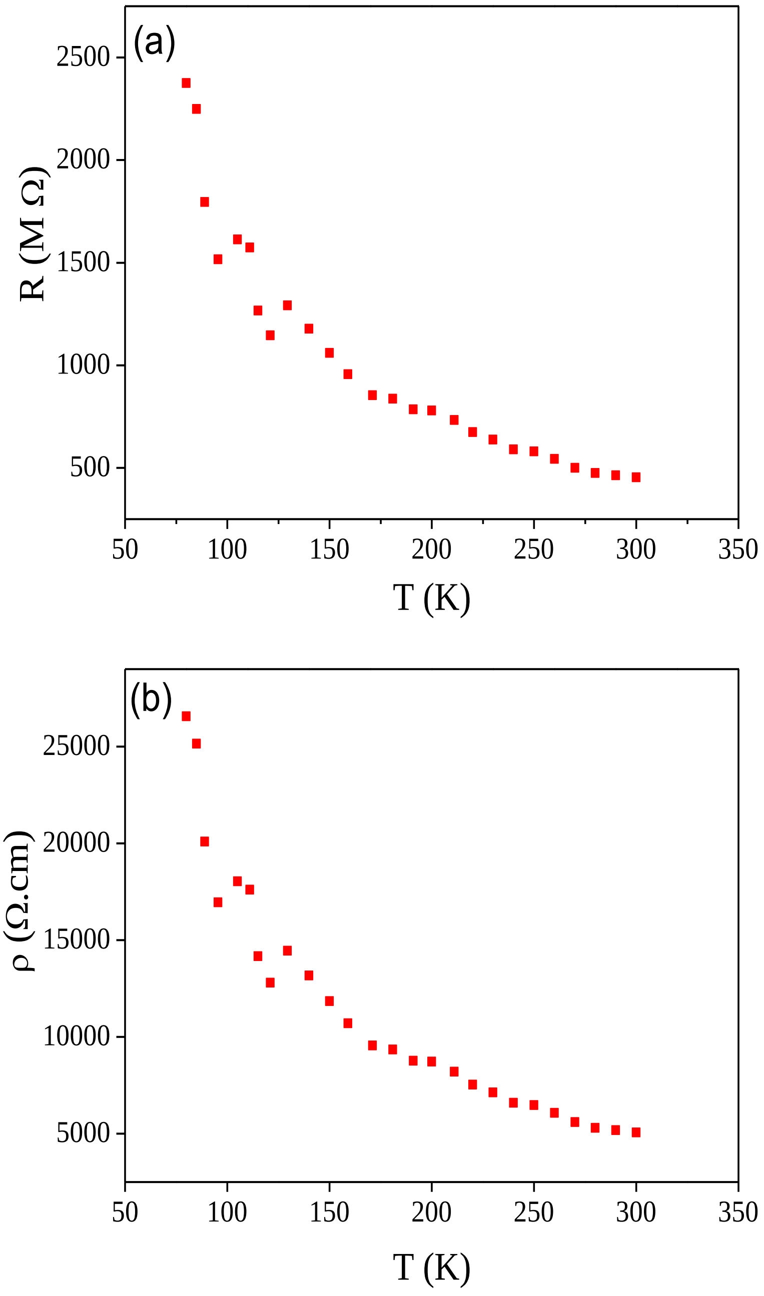

Figure S26. (a) Plot of electrical resistance of Cd^{2+}-**NDS1** NW (L2) as a function of temperature from 80 – 300 K and (b) Plot of static resistivity of Cd^{2+}-**NDS1** NW (L2) as a function of temperature from 80 – 300 K.

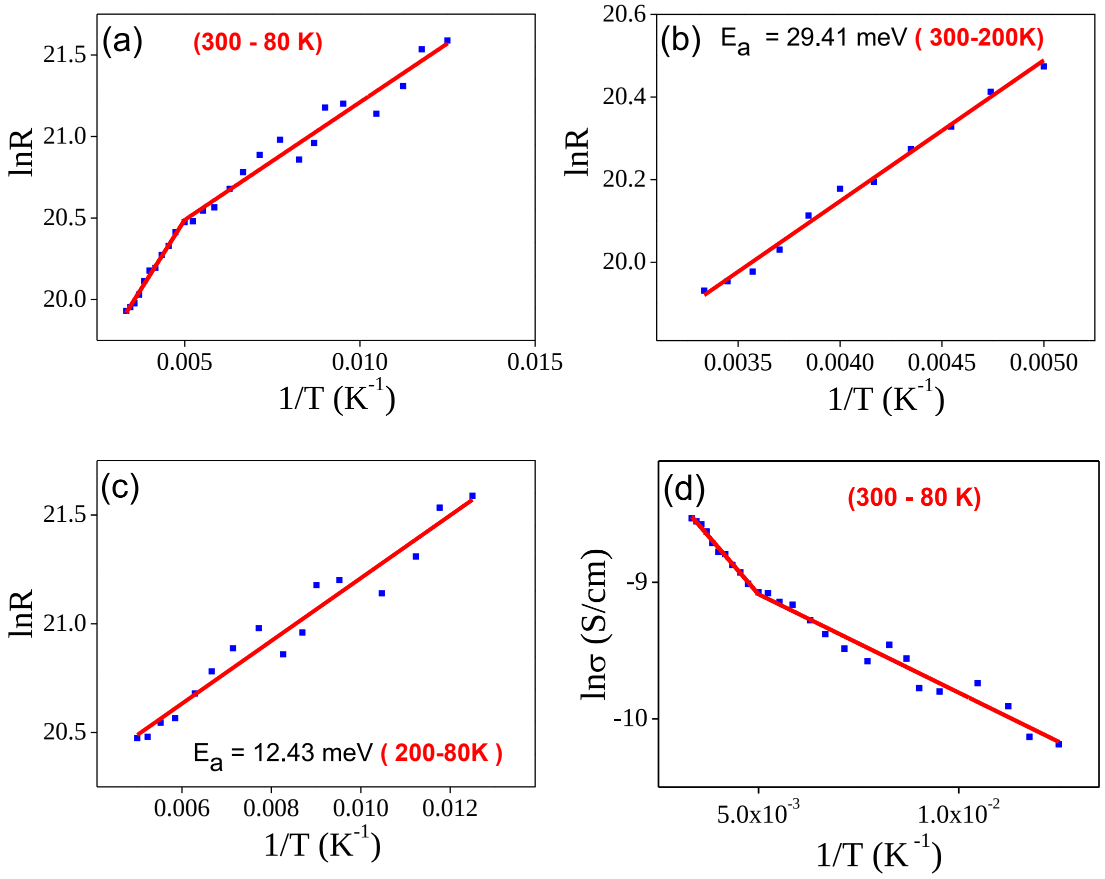

Figure S27. (a-c) Activation energy (E_a) calculations from "lnR *Vs* 1/T" plot based on Arrhenius equation $\mathbf{R = R_0}\ \mathbf{\mathit{exp}^{(Ea/kT)}}$ and (d) Plot between "lnσ Vs 1/T" to support the activation energy (E_a) calculations.

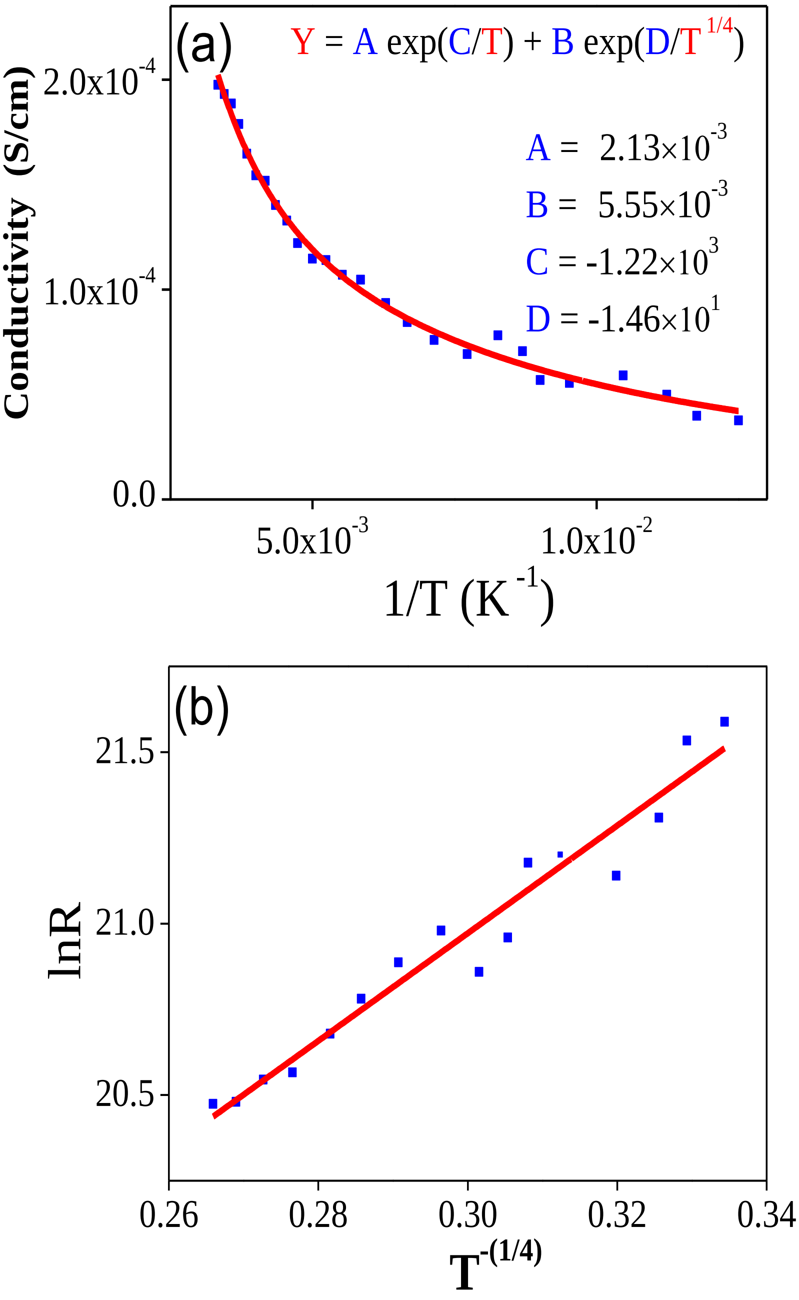

Figure S28. Plots of "Conductivity *Vs* 1/T" and "lnR Vs $T^{(-1/4)}$" for evaluation of electron transport mechanisms in Cd^{2+}-**NDS1** NW (L2) conductivity by nonlinear and linear fittings, respectively.

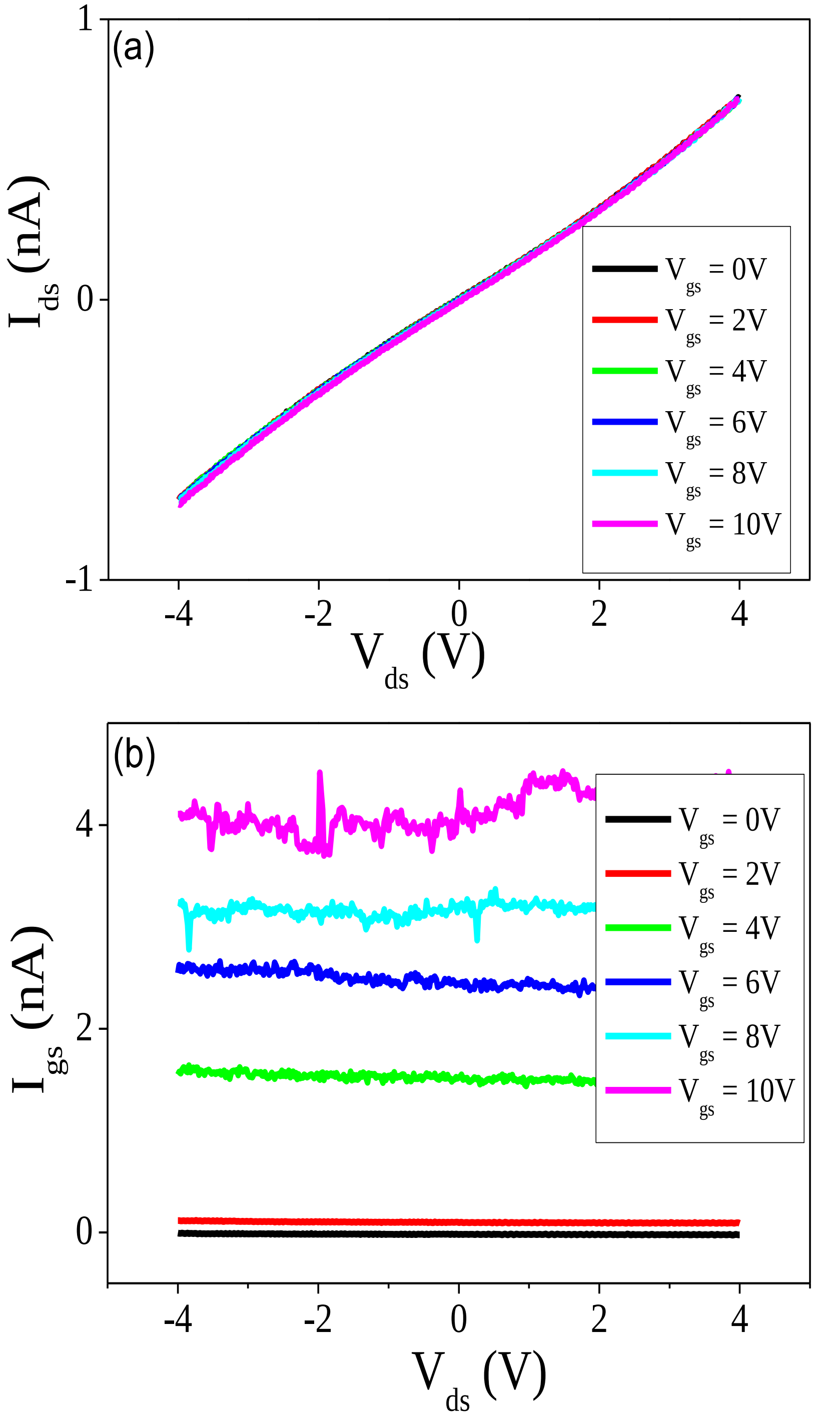

Figure S29. (a) Drain current (I_{ds}) versus applied drain voltage (V_{ds}) and (b) Gate leakage current versus applied drain voltage (V_{ds}) for L1 scanned between -4 to 4 V with varied gate voltage (V_{gs}) ranged from 0 to 10 V.

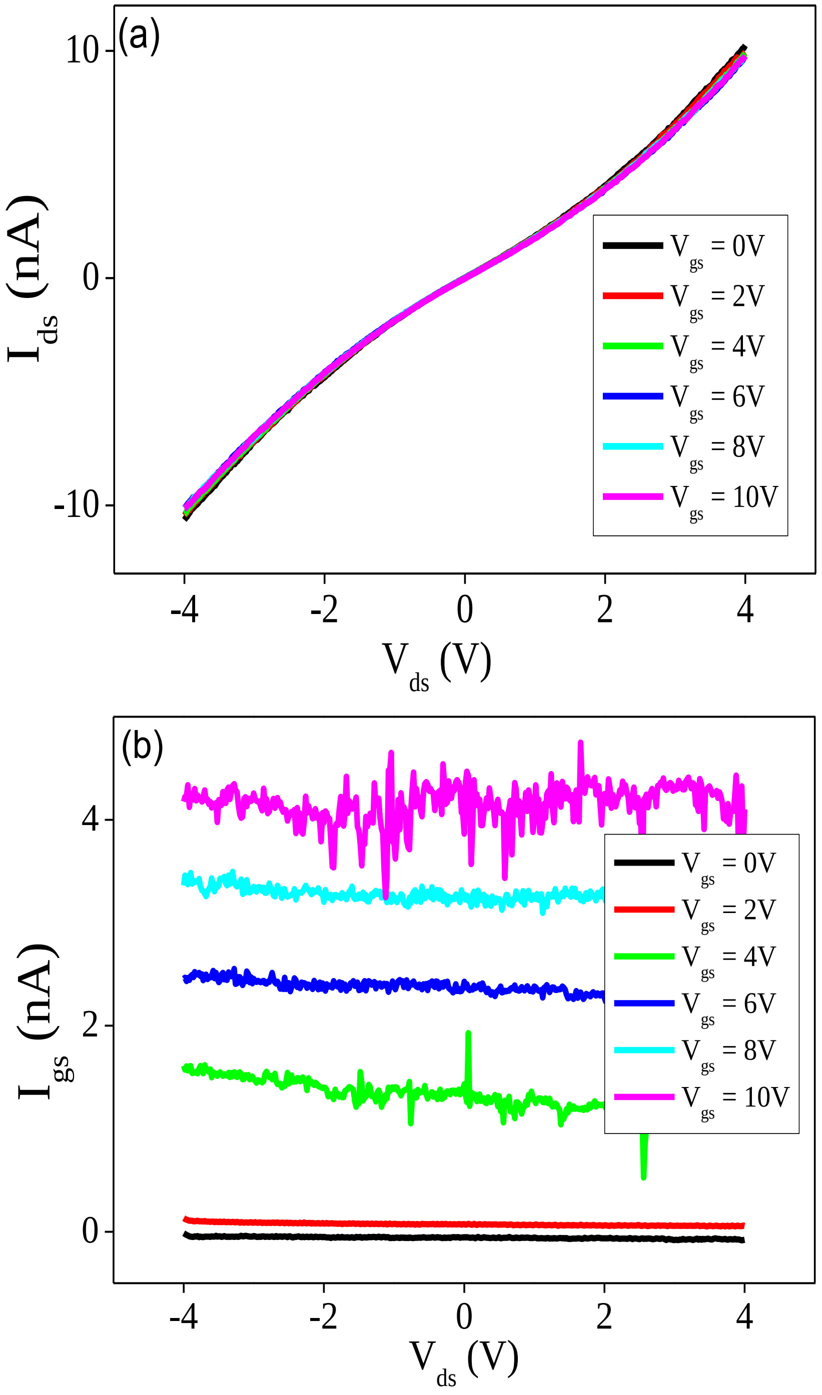

Figure S30. (a) Drain current (I_{ds}) versus applied drain voltage (V_{ds}) and (b) Gate leakage current versus applied drain voltage (V_{ds}) for L2 scanned between -4 to 4 V with varied gate voltage (V_{gs}) ranged from 0 to 10 V.

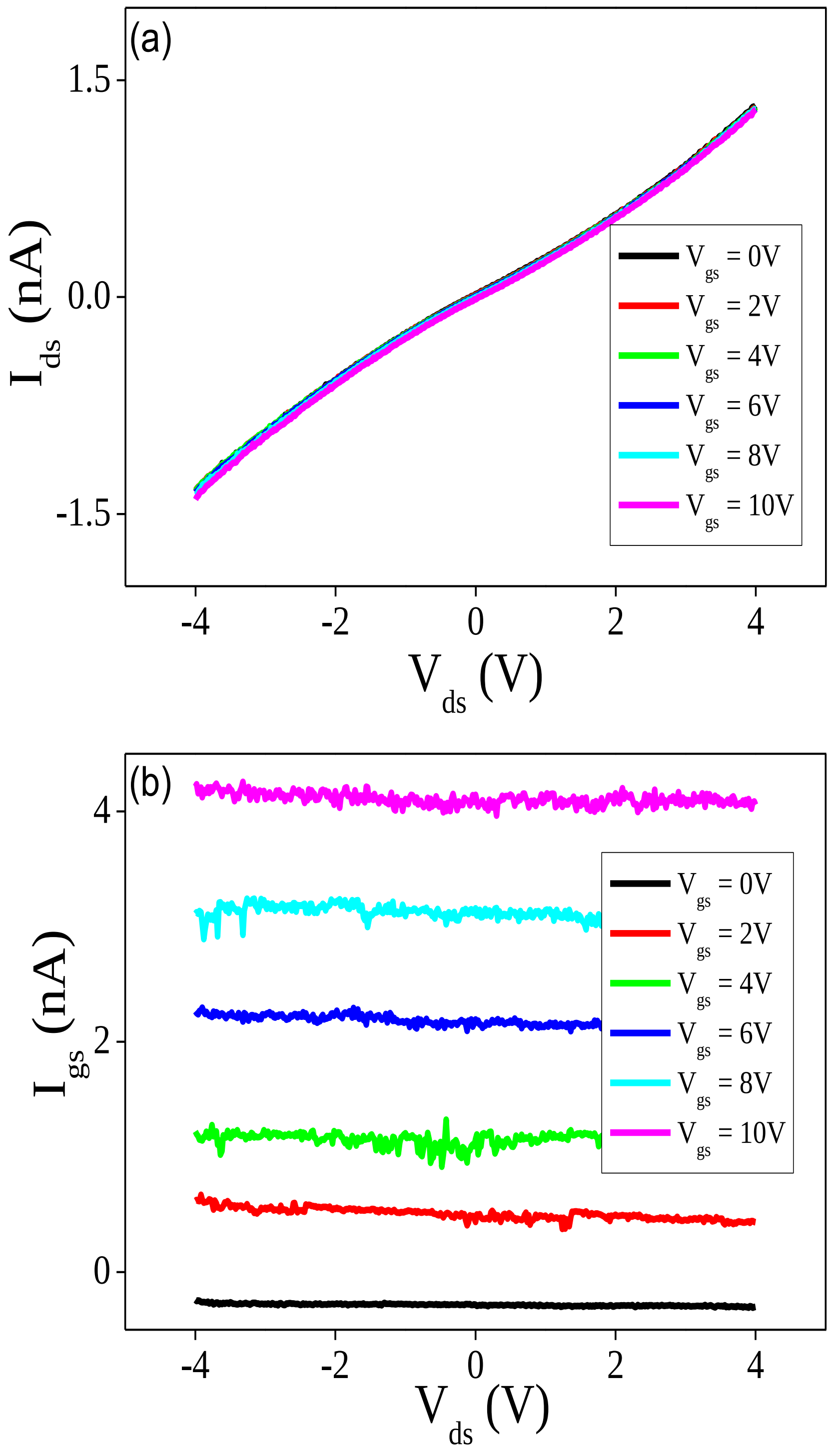

Figure S31. (a) Drain current (I_{ds}) versus applied drain voltage (V_{ds}) and (b) Gate leakage current versus applied drain voltage (V_{ds}) for L3 scanned between -4 to 4 V with varied gate voltage (V_{gs}) ranged from 0 to 10 V.
